# The Radiation-Transmission-Reception (RTR) model of propagation: Implications for the effectiveness of network interventions

**DOI:** 10.1371/journal.pone.0207865

**Published:** 2018-12-05

**Authors:** Wouter Vermeer, Otto Koppius, Peter Vervest

**Affiliations:** 1 Northwestern Institute for complex systems (NICO), Northwestern University, Evanston, IL, United States of America; 2 Center for Prevention Implementation Methodology (Ce-PIM), Feinberg School of Medicine, Northwestern University, Chicago, IL, United States of America; 3 Center for Prevention Implementation Methodology (CCL), School of Education and Social Policy, Northwestern University, Evanston, IL, United States of America; 4 Department of Technology & Operations Management, RSM Erasmus University, Rotterdam, The Netherlands; Columbia University, UNITED STATES

## Abstract

Propagating phenomena in networks have received significant amount of attention within various domains, ranging from contagion in epidemiology, to diffusion of innovations and social influence on behavior and communication. Often these studies attempt to model propagation processes in networks to create interventions that steer propagation dynamics towards desired or away from undesired outcomes. Traditionally, studies have used relatively simple models of the propagation mechanism. In most propagation models this mechanism is described as a monolithic process and a single parameter for the infection rate. Such a description of the propagation mechanism is a severe simplification of mechanisms described in various theoretical exchange theories and phenomena found in real world settings, and largely fails to capture the nuances present in such descriptions. Recent work has suggested that such a simplification may not be sufficient to explain observed propagation dynamics, as nuances of the mechanism of propagation can have a severe impact on its dynamics. This suggests a better understanding of the role of the propagation mechanism is desired. In this paper we put forward a novel framework and model for propagation, the RTR framework. This framework, based on communication theory, decomposes the propagation mechanism into three sub-processes; Radiation, Transmission and Reception (RTR). We show that the RTR framework provides a more detailed way for specifying and conceptually thinking about the process of propagation, aligns better with existing real world interventions, and allows for gaining new insights into effective intervention strategies. By decomposing the propagation mechanism, we show that the specifications of this mechanism can have significant impact on the effectiveness of network interventions. We show that for the same composite single-parameter specification, different decompositions in Radiation, Transmission and Reception yield very different effectiveness estimates for the same network intervention, from 30% less effective to 70% more effective. We find that the appropriate choice for intervention depends strongly on the decomposition of the propagation mechanism. Our findings highlight that a correct decomposition of the mechanism is a prerequisite for developing effective network intervention strategies, and that the use of monolithic models, which oversimplify the mechanism, can be problematic of supporting decisions related to network interventions. In contrast, by allowing more detailed specification of the propagation mechanism and enabling this mechanism to be linked to existing interventions, the RTR framework provides a valuable tool for those designing interventions and implementing interventions strategies.

## Introduction

Propagating processes underlie many phenomena we observe in our daily lives; we are constantly influenced socially, form opinions based on those with whom we interact, process information from various sources to shape our view of our world, and are fighting off viral threats in our environment. Academia has long recognized the value of using a network lens to study such phenomena. Consequently, network analysis has been a cornerstone in domains such as epidemiology [1–3], social contagion [4–6], intervention dissemination and implementation [7], information diffusion, [8] and learning [9, 10]. While the phenomena in each of these domains differ from one another, they collectively belong to a generic class of propagation processes. A propagation process we define as *‘a process by which the behavior or state of an agent (A) will result in a change of behavior or state of connected neighbor (B)’*.

Adopting a network lens has contributed to highlighting the role of the network structure in driving the dynamics of propagating processes. For example, this body of work has better informed us on the relevance of characteristic path length [11], clustering [12, 13], and network topologies [11, 14]. In particular it has been found that most social systems contain degree distributions with long tails or scale-free structures [14–16], and that nodes with high degree facilitate spread [17–19]. Our social systems, combining these two features, are prone to be affected by large propagating events, or cascades (of failure) [20]. Additionally, due to increasing globalization and growing interconnectedness of our society, such cascades are likely to become more profound and frequent in the future. The 2014 Ebola outbreak, the 2010 Arab Spring, or the 2008 financial crisis, are but a handful of recent high profile examples that reveal the potential impact such propagating cascades can have, and these events signal the high societal and scientific need for understanding how cascading dynamics come about.

A large body of work has focused on improving this understanding, and in doing so has mainly highlighted the network structure and topology as the primary driver of propagation dynamics. Similarly, embracing the path dependance that is inherent to many social phenomena, a large body of work has focused on the effects of local structures, in particularly surrounding the seed of the propagation process (the location where in the propagation process originates from). Many network intervention strategies are now aimed at finding the best positioned individuals to increase the momentum and reach of spreading phenomena [21–23]. While this body of work has made a strong case for network structure as a critical factor determining propagation dynamics, in identifying such effects it has largely ignored the impact that different mechanisms of propagation—the way in which propagation process takes place—can have, as propagation processes are generally treated as a class of uniform processes. While recognizing the importance of the network structure for understanding propagation dynamics, we argue that it is not network structure alone that drives propagation dynamics, and that fully understanding these dynamics requires one to consider the propagation mechanism.

In studies on propagation phenomena, the mechanism has generally been implemented as a single rate or probability which summarizes the success rate of the propagation process. Yet many theories, e.g. social exchange theory [24], communication theory [25], and complex contagion [26], and more recent studies in marketing [4, 27] and biological systems [28] have suggested that the mechanism of propagation in reality can be much more complicated. For example, recent marketing studies have incorporated individual level characteristics that describe how likely someone is to spread or how susceptible one is to marketing, highlighting how the mechanism can be different depending on the pair of agents that is considered, making it locally bound. Note that propagation literature has painted a relatively clear picture on the impact of network structure, and based on this has developed various targeting strategies, yet it remains largely unclear how (variations in) the propagation mechanism would affect propagation dynamics and thus our knowledge of how cascades occur and can be influenced.

To address this gap, in this paper we propose a new framework for propagation which incorporates a more nuanced notion of the propagation mechanism. Based on a framework from communication theory [25] we consider the propagation mechanism to be composed of three different sub-processes; ***R***adiation, the process by which an agent sends out a signal, ***T***ransmission, the process by which that signal reaches the alter, and ***R***eception, the process by which the signal is processed by the alter. We will show that decomposing the mechanism in this way not only provides a more realistic way of capturing the propagation process and aligns with existing intervention strategies, but also that it provides new insight into *how* to more effectively intervene in propagation phenomena once they occur.

## Propagation in networks

The definition of propagation describes it as dyadic process of influence between two agents (*‘a process by which the behavior or state of an agent (A) will result in a change of behavior or state of connected neighbor (B)’*). It highlights the initial shock at one agent that puts in motion the consequent effect at another agent, making propagation effectively a process that should be considered on the agent level. Note that the definition of propagation stays void of any reference to a propagation mechanism. Therefore we leverage a perspective from communication theory to infer the propagation mechanism. Communication itself is an instance of propagation that describes the exchange of information between agents. However, whereas for propagation processes the mechanism is obscured, for the communication process the mechanisms has been made explicit and often been the focus of study. For decades communication theory [25] has been applied to describe the dyadic mechanism of communication.

In describing the information exchange process, communication literature identifies a mechanism of exchange using three entities, each with their own role; 1) a sender that encodes information, 2) a medium that transports this information (from sender to receiver), and 3) the receiver that decodes the information. It highlights the agency of each of these entities, and the potential of loss of information during each of their actions. As such, the communication perspective considers the propagation mechanism as an agent-driven process consisting of three consecutive steps. Applying this lens to other propagation phenomena allows one to identify these steps across the complete spectrum of propagation phenomena (e.g. [29]), suggesting the decomposition of propagation into three sub-processes to be broadly applicable.

**Fig 1:**
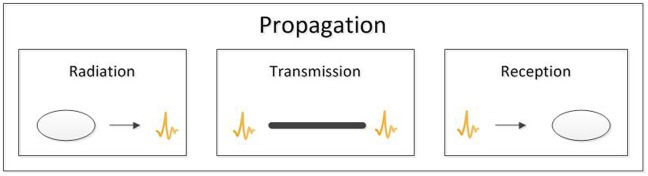
Propagation is composed of three distinct sub-processes; Radiation refers to the process with which an actor translates a change in state into an outgoing signal, Transmission to the process by which this signal is transferred (via a tie) towards the alter(s), and Reception to the process by which alters respond to the incoming signal and may change state accordingly.

Following this observation, we formulate a framework of the propagation mechanism using three sub-processes (Fig 1).

We modify the communication-theoretical framework in two ways. First, given our goal of describing propagation phenomena that go far beyond the communication setting, we step away from communication-specific terminology, and use the labels Radiation, Transmission and Reception (RTR) for describing the sub-processes during propagation. *Radiation* refers to the process by which a change in the state or behavior of an actor results in that actor sending out signals to its outgoing ties. *Transmission* refers to the process by which a signal is transferred from the sender to the receiver (via a tie). *Reception* refers to the process by which actors respond to the incoming signal(s) and change their behavior or state accordingly. Like in communication theory, these processes are sequential, meaning that radiation is required for transmission and transmission is required for reception. Only when all three sub-processes have taken place successfully one can speak of propagation.

Second, we step away from the purely dyadic notion of propagation, and consider it to be an (agent-based) network process. Each of the sub-processes can be observed to adopt a different logic: Radiation is sender-based, and a process from one actor to its outgoing ties, making it potentially a one-to-many process. Transmission is medium-based, and occurs within a single tie and hence is a dyadic process. Reception is receiver-based, and is a process potentially from many incoming ties to one single actor, making it effectively a many-to-one process. These differences highlight that while all sub-processes are part the composite propagation process, each of them is conceptually different from one another. Furthermore, it shows that while each dyad with propagation will require all three sub-processes to occur, the propagation process itself is fundamentally a network process.

Revisiting the literature on propagation with the RTR framework in mind provides some interesting insights (Table 1). First, to our knowledge, there are no studies that cover all three sub-processes simultaneously. Second, each of the sub-processes is grounded in literature, examples of each of them individually can be found in previous work. Third, as an integrated framework for sub-processes has been missing, the labeling in current literature has proven to be inconsistent. A single term has been used to refer to different parts of the mechanism in different articles, and different terms are used across articles for parts of the mechanisms that are in fact identical. Fourth, propagation is often considered a one-sided process (rather than a dyadic one). It is mostly described as a process by which agents change state/behavior, and thus resembles the reception sub-process. Lastly, lessons learned and conclusions drawn in propagation literature are generally assumed to translate across settings, disregarding the potential impact of the propagation mechanism adopted, as such sub-processes have effectively been considered to be inter-changeable.

**Table 1:** This table depicts some examples of the way in which propagation has been operationalized in literature. Different streams of literature focus on different sub-processes, and thus capture only part and not the whole propagation process.

Author	Year	Label of propagation	Context	Sub-process considered		
				Radiation	Transmission	Reception
Granovetter [30]	1973	Diffusion	Information (job opportunities)		x	
Rogers [8]	1995	Diffusion	Innovation (general)			x
Szulanski [31]	1996	Transfer	Knowledge transfer (best practices)	x		x
Reagans and McEvily [32]	2003	Transfer	Knowledge transfer (R&D)			x
Dodds and Watts [33]	2004	Contagion	Epidemics / Social behavior (general)			x
Valente [34]	2005	Diffusion	Innovation (general)	x		x
Buzna et al. [35]	2006	Spreading	Infrastructure (general)			x
Ferguson et al. [36]	2006	Transmission	Epidemics (influenza)			x
Centola and Macy [26]	2007	Contagion	Social behavior (general)	x		x
Rahmandad and Sterman [13]	2008	Diffusion	Epidemics (general)	x		
Buldyrev et al. [20]	2010	Cascades	Infrastructure (general)		x	x
Iyengar et al. [37]	2010	Contagion	Innovation (medical drug)			x
Gonzalez-Bailon et al. [38]	2011	Diffusion	Social behavior (protests)			x
Birken et al. [39]	2012	Influence	Innovation (healthcare practice)	x		
Aral et al. [40]	2013	Influence	Innovation (mobile app)	x		x
Banerjee et al. [41]	2013	Contagion	Innovation (micro finance)	x		x
Falk et al. [42]	2013	Influence	Innovation (tv shows)	x		x
Barasch and Berger [43]	2014	Sharing	Information (general)	x		
Smilkov et al. [44]	2014	Spreading	Epidemics (general)			x
Contractor and DeChurch [45]	2014	Influence	Innovation (medicine)	x		x
Goel et al. [46]	2015	Diffusion	Information (tweets)	x		
Pei et al. [47]	2015	Diffusion	Information (blogs)		x	
Sutton et al. [48]	2015	Diffusion	Information (tweets)			x
Valdez et al. [49]	2015	Spreading	Epidemics (ebola)	x	x	
Bardoscia et al. [50]	2016	Contagion	Finance (financial distrss)	x		
Canini et al. [51]	2016	Transmission	Epidemics (influenza)	x		
Fu et al. [52]	2017	Contagion	Epidemics (influenza)			x
Wang et al. [53]	2017	Spreading	Epidemics (general)			x
Wang et al. [28]	2017	Transmission	Gene behavior (general)	x		x

Our exploration of the literature indicates that the propagation mechanism, the process that describes how propagation between two (or more) agents occurs, has received relatively little attention in propagation literature. In most studies, the mechanism of propagation adopted is a direct consequence of the model type being used. Most commonly adopted types of models are (variation or extensions of) of SIS/SIR model [54], threshold models [8, 55], cascade models [56], or Bass(-like) models [57]. Each of these models has a slightly different mechanism of propagation, yet all of them are unified in their assumption that propagation is a monolithic process.

Recent work [26, 28, 40, 41] has started to make a case for stepping away from a monolithic view of the propagation mechanism to better understand propagation dynamics. Centola & Macy [26] in their study on complex contagion, a spreading mechanism which assumes an agent requires stimuli from multiple sources to change state, has shown that both the speed and pervasiveness of propagation are strongly influenced by such a variation in propagation mechanism. Similarly, [28, 40, 41] highlight that agents can send out very different strengths of signals and/or can be susceptible to different magnitudes of signals [28, 40], and that such heterogeneity in the mechanism has a strong influence on the dynamics of propagation. By incorporating variations in the propagation mechanism, these studies highlight the marked effect the mechanism can have on the dynamics of the propagation process.

Although these examples have adopted more complex models to be able to capture nuances in the propagation mechanism, there is still limited effort in making sure these more complex models can be related to each other. Therefore, propagation literature runs the risk of entering an era in which highly specific mechanisms are being used with limited contributions to generalizable knowledge. The RTR-framework addresses this tension and allows us to build a model which can be used to unify the various propagation models (including more complex ones), and make explicit how mechanisms differ (or align). This model thus facilitates the propagation field to move to a place in which the impact of the propagation mechanism can be structurally studied and better understood.

The RTR-model for propagation, in line with the RTR-framework, specifies the propagation mechanism using three sub-processes, each using the own function. Each of them can be tailored depending on the needs of a specific setting and phenomena. For simple scenarios these functions can be reduced into three single parameters (one for each sub-process) that capture the success of the specific sub-process; *Radiation* (*α*), *Transmission* (*ϕ*), and *Reception* (*η*), making the composite transmission likelihood (λ) equal to *α* × *ϕ* × *η*. For more complex scenarios these function can be extended to include memory effects [58], threshold behavior [55], and complex contagion effects [26] (a full model specification can be found in S3 File).

## Steering propagation outcomes

For many propagation phenomena, understanding what drives dynamics is only a first step. Generally, one wants to affect and steer the outcomes of the propagation process using one’s knowledge of the process. For example, we want to understand disease spread so we can prevent it, we want to understand opinion dynamics so we can influence them, and we want to understand word-of-mouth so we can market our products better. There are various streams of literature aimed at understanding propagation dynamics. Some have focused on identifying the controllability of network structures [59], others have focused on the predictability of large scale cascades [60], and yet others have tested the impact of interventions [21, 61, 62]. Each of these streams has focused on a different dimension of understanding propagation dynamics, yet all strive towards the goal of increasing our knowledge of propagation dynamics in the hopes of improving our ability to intervene and more effectively steer such dynamics. As such, understanding dynamics goes hand in hand with intervening in them.

Valente [62] provides an overview of network interventions, and classifies four distinct categories of interventions in networks: Individuals, Segmentation, Induction, and Alteration. Interventions focused on individuals revolve around identifying key individuals as targets for intervention, these can either be individuals who are very efficient in getting a message out, or low-threshold change agents, those who will easily change their behavior/state, or those in favorable network positions. Segmentation interventions aim to leverage the partitioning of a system in clusters in order to facilitate maximum reach, and thus selects key individuals based on system-level structure. Induction interventions highlight the relevance of the multiplex nature of networks and aim to leverage existing structure beyond their normal use, ie. use word-of-mouth (WOM) in social networks to facilitate product adoption. Alteration interventions aim to change network structure, by adding or removing links or agents, in such a way that spreading will become more (or less) likely. Based on this classification, three factors that affect the impact of interventions can be identified; 1) agent level characteristics that affect the mechanism by which propagation occurs, 2) the network structure, and 3) the location within the network that is being affected. These three factors seem to address *where* to target interventions, yet leave the question *how* to (most effectively) intervene largely unanswered. Valente recognized this and states *“because network interventions are based on accelerating social influence, they require a deeper understanding of the social mechanisms driving behavior change”* ([62], page 52), suggesting that understanding impact of the mechanism of propagation might provide an avenue to address how to best intervene.

The monolithic mechanism that is adopted traditionally in propagation literature and models allows for only a single type of intervention—a change of the rate (or success) of propagation. While relevant lessons have been learned from studying such interventions, e.g. that the epidemic threshold varies based on network topology [19, 63, 64], it leaves little room for exploring how to best intervene, and thus provides little insight in how to best devise intervention strategies. Comparing various types of interventions and devising intervention strategies requires a framework in which effects of different types of interventions can be disentangled and distinguished. The RTR framework of propagation, by decomposing the mechanism into three sub-processes, does exactly this.

From a practical perspective, the three sub-processes identified in the RTR-model of propagation align naturally with interventions. An overview of interventions made in practice to improve/reduce spreading in various contexts (S2 File) shows that such interventions can be easily translated into changes within specific single sub-process. Interventions thus seem to map directly to changes in the mechanism. Combining this with the notion that propagation dynamics are driven by the propagation mechanism (in combination with the network structure), this makes effective intervening a question of understanding how interventions change the mechanism, and how this consequently translates into changed propagation dynamics. This question can only be addressed when a model of propagation is adopted that incorporates a more detailed view of the mechanism of propagation.

Similarly, from a conceptual standpoint, we find that the classification of network interventions [62] aligns well with the RTR framework of propagation. Individual type of interventions based on individual characteristics can be directly linked to specific sub-processes; selecting individuals who are able to communicate well is an intervention that aims to leverage the strong capacity for sending out signals, and thus can be considered a radiation-based intervention. Selecting low-threshold agents who easily change their state or behavior would be a reception-based intervention, and both induction and alteration type interventions (which focus on enabling spread by improving the structure that underlies transfer) can be considered transmission-based an intervention. The RTR framework thus provides a structured way to distinguish between these network interventions. It should be noted that segmentation type interventions are an outlier in this aspect. As such interventions consider system-wide network structural properties (rather than anything mechanism related) such effects are nearly impossible to capture using an agent-level description of the propagation mechanism.

Interventions in traditional propagation models, which are generally monolithic, do not allow for specific parts of the mechanism to be targeted. Consequently, in such setting the various types of interventions are hard to be distinguished and are generally considered to be inter-changeable. The fact that the sub-processes are conceptually different, and can be associated to specific interventions, suggests that there are conceptual differences in intervention that cannot be captured using traditional models, and are therefore overlooked in current propagation research. For this reasons we argue that models incorporating a more nuanced view of the propagation mechanism—like the RTR model presented in this paper—are required for effectively studying network intervention effectiveness and intervention strategies.

## Materials and methods

The RTR framework describes the mechanism of propagation, allowing a change in state to propagate from one actor to affect its connected neighbors (alters) using three distinct sub-processes. The model that captures the propagation mechanism can therefore be described by means of three separate functions: one for Radiation, one for Transmission, and one for Reception. The model described below is the most generic form of the model. It allows for capturing both stochastic and deterministic processes, discrete and continuous agent states, more complex non-linear propagation dynamics [26] and memory effects [33, 58]. The radiation function describes how the change in state of an agent *i* (Δ*s*_*i*,*t*_), if higher than the radiation threshold (*u*), will result in signals being sent ($p_{i,e,t}^{out}\in P_{i,t}^{out}$) to the outgoing ties ($E_{i}^{out}$).

$$\begin{array}{c} \sum_{t^{\prime }=t-T^{rad}+1}^{t}\sum \left(A_{i,t^{\prime }}\times P_{i,t^{\prime }}^{out}\times {\left(\tau_{rad}\right)}^{(t-t^{\prime })+1}\right)=\{\begin{array}{cc} \Delta s_{i,t} & \;\text{if}\;\Delta s_{i,t}\geq u\\ 0 & \;\text{if}\;\Delta s_{i,t}<u \end{array} \end{array}$$ (1)

In which *A*_*i*,*t*_ (*α*_*i*,*e*,*t*_ ∈ *A*_*i*,*t*_) is a vector capturing the characteristics which influence the radiation per outgoing edge *e* at time *t*, *T*^*rad*^ is the radiation memory duration, i.e. the number of time units after a change of state that this change can cause radiation, and *τ*_*rad*_ is the memory inflation factor, the extent to which past changes are amplified or dampened.

The transmission function describes how the signal radiated ($p_{i,e,t}^{out}\in P_{i,t}^{out}$) is transformed into the signal which is received by the alter *j* ($p_{e,j,t}^{in}$)

$$\begin{array}{c} p_{i,e,t}^{out}=\sum_{t^{\prime }=t-T^{tra}+1}^{t}\left(\phi_{e,t^{\prime }}\times p_{e,j,t^{\prime }}^{in}\times {\left(\tau_{tra}\right)}^{(t-t^{\prime })+1}\right) \end{array}$$ (2)

In which *T*^*tra*^ is the memory duration for transmission, *τ*_*tra*_ is the memory inflation factor for transmission, and *ϕ*_*e*,*t*_ is a vector of edge characteristics which influence transmission.

The reception function describes how transmitted signals ($p_{e,j,t}^{in}$) received from the incoming ties ($E_{j}^{in}$) translate into a change of state of an alter if their combined effect passes the reception threshold (*q*).

$$\begin{array}{c} \Delta s_{j,t}=\{\begin{array}{c} \sum_{t^{\prime }=t-T^{rec}+1}^{t}\sum \left(P_{j,t^{\prime }}^{in}\times \Psi_{j,t^{\prime }}\times {\left(\tau_{rec}\right)}^{(t-t^{\prime })+1}\right)\;\text{if:}\;\\ \sum_{t^{\prime }=t-T^{rec}+1}^{t}\sum \left(P_{j,t^{\prime }}^{in}\times \Psi_{j,t^{\prime }}\times {\left(\tau_{rec}\right)}^{(t-t^{\prime })+1}\right)\geq q\\ \;\;\;\;\;\;\;\;\;\text{or}\;\\ 0\;\;\text{if:}\;\;\sum_{t^{\prime }=t-T^{rec}+1}^{t}\sum \left(P_{j,t^{\prime }}^{in}\times \Psi_{j,t^{\prime }}\times {\left(\tau_{rec}\right)}^{(t-t^{\prime })+1}\right)<q \end{array} \end{array}$$ (3)

In which *T*^*rec*^ is the memory duration for reception, *τ*_*rec*_ is the memory inflation factor for reception, and Ψ_*j*,*t*_ (*η*_*e*,*j*,*t*_ ∈ Ψ_*j*,*t*_) is a vector capturing actor specific characteristics influencing the reception per incoming edge *e* on time *t*. Using Eqs 1, 2 and 3, the RTR-model decomposes the propagation process into three distinct sub-processes.

### The study design

The generic RTR model allows for capturing the full range of propagation mechanisms and thus provides much more flexibility compared to traditional propagation models. Note that such flexibility comes at the cost of a more complex model, a tradeoff that is generally hard to balance. Rather than making a statistical argument for more (or less) complex models of propagation, our approach is to show the value of decomposing the propagation process (in RTR) from an intervention perspective as this highlights the different affordances of the RTR model. We do so for a relatively simple well-studied mechanism, the mechanism that is traditionally used in SIS models in epidemiology.

The generic RTR model can be aligned with the SIS model by making a set of restricting assumptions relating to the propagation mechanism. First, it is assumed that memory plays no role, second, that agent states are binary (infected/susceptible), third, that the mechanism of propagation is stochastic, and lastly, that infected agents can infect others as long as they remain in the infected state (effectively making radiation a function based on the state rather than a change in state). Incorporating these assumptions into the RTR model, the generic model can be rewritten as in the much simpler version below. Details on how the generic model reduces to the SIS/SIR and other traditional models such as Bass(-like) models [57], threshold models [8, 55] and cascade models [56] are provided in S3 File.

$$\begin{array}{c} p_{i,e,t}^{out}=\{\begin{array}{cc} ∼Bern(\alpha_{i,e,t}^{*}) & \;\text{if}\;s_{i,t}=1\\ 0 & \;\text{if}\;s_{i,t}=0 \end{array} \end{array}$$ (4)

$$\begin{array}{c} p_{e,j,t}^{in}∼Bern\left(\phi_{e,t}^{*}\right)\;\text{if}\;p_{i,e,t}^{out}=1 \end{array}$$ (5)

$$\begin{array}{c} \Delta s_{j,t}=\{\begin{array}{cc} ∼Bern(1-\prod \left(1-\Psi_{j,t}^{*}\right))\; & \text{if}\;s_{i,t}=0\\ 0\; & \text{if}\;s_{i,t}=1 \end{array} \end{array}$$ (6)

Then if one assumes homogeneous agents, no interaction effects among signals sent or received, an unweighted network structure, and a stable propagation mechanism over time ($\alpha_{i,e,t}^{*}=\alpha$, and $\phi_{e,t}^{*}=\phi$, and $(1-\Psi_{j,t}^{*})=\eta$) one can reduce propagation on the dyadic level (between two agents) to λ = ∼*Bern*(*α* × *η* × *ϕ*).

Note that the only difference between traditional SIS model and this version of the RTR model is the that traditional model assumes the sub-processes to be inter-changeable (and thus the same), whereas the RTR model allows them to be independent. A comparative simulation experiment with the above-stated SIS version of the RTR model is done to explore the implications of this assumption under various interventions.

The experimental setup is as follows: four scenarios with the same exact composite propagation rate/probability (λ = *α* × *η* × *ϕ*) are selected. One among them will be a scenario in which sub-processes are indistinguishable, meaning that each sub-process has the exact same parameter value. This operationalization can be reduced to a single parameter mechanism which captures the traditional SIS behavior, and will be considered the baseline to which behavior is compared. The other three scenarios use different decompositions in sub-processes, while still maintaining the exact same composite propagation rate (λ) as the baseline process with identical sub-processes. The decomposition of the four scenarios in our experiment can be found in Table 2.

**Table 2:** Four scenarios with varying decomposition of the sub-processes of propagation.

Scenario	Composite propagation probability (λ)	Radiation probability (*α*)	Transmissio probability (*ϕ*)	Reception probability (*η*)
Scenario 1	0.192	0.4	0.6	0.8
Scenario 2	0.192	0.6	0.8	0.4
Scenario 3	0.192	0.8	0.4	0.6
Scenario 4 (baseline)	0.192	0.5769	0.5769	0.5769

All scenarios have the same composite propagation probability (λ = *α* × *η* × *ϕ* = 0.192). This probability is however decomposed differently across the sub-processes for each of the scenarios.

The RTR model describes the propagation mechanism on the agent level and is therefore implemented as an Agent-Based Model (ABM). It has been shown [13] that, due to the discrete nature of Agent-based models (ABMs), these models yield slightly different behavior compared differential equation models (DEMs) traditionally used to describe SIS spreading dynamics. In particular, small infection sizes can be affected by the discrete nature of an ABM, yielding extinction events in ABMs that will not occur in DEMs. To avoid the effect of premature extinction events the recovery rate in our simulations is set such that *R*_0_ is well above 1 (*ρ* = 0.2 → *R*_0_ = 2.88), which makes extinction highly unlikely. Consequently our scenarios are expected to yield widespread cascades of infections. Additionally, should any early extinction event occur, their results are excluded from the analysis.

As previously indicated both the network structure and seed chosen can have strong effects on the propagation dynamics. As we aim to show the impact of the mechanism of propagation both of these effects will need to be controlled for during the experiment.

We control for the impact of network structure by keeping it constant across simulations. While this does not erase the impact of network structure, it does keep its effects constant across simulations, and thus allows variations in dynamics to be attributed to variations in the mechanism of propagation. Additionally, to make sure our results are generalizable, we run our experiments on three network structures.

The first network, which will be used throughout this paper as the setting in which to present results, is an undirected, non-weighted, scale-free network synthetically generated based on the Barabasi-Albert approach [14]. It has has 10.000 nodes (*n* = 10.000) and an average degree of 3 (*m* = 3). The second network used is a Facebook Ego-network [65]. It covers a giant component of friendship ties, based on the social circles on Facebook platform, with *n* = 4.039 and *e* = 88.234, and is thus denser than the original synthetic network. The Third network is the Autonomous systems AS-733 network [66]. It describes the communication among a set of autonomous systems, and is based on BGP (Border Gateway Protocol) logs. This network consists of 6.474 nodes and 12.572 edges and is thus sparser then our synthetic network.

We control for the impact of the seeding strategy by considering two seeding strategies. We will use random seeding as the default strategy, in this strategy a single seed is randomly selected for each simulation. To control for the impact of seeding we also adopt a second strategy which we label the betweenness strategy, in this strategy seeding is ‘optimized’ by selecting the actor with the highest betweenness centrality as the seed in each simulation. To further increase the reliability of our results, each setting will be bootstrapped 10000 times, and the mean behavior across this set of bootstrapped iterations is then used in our analysis.

Following the mapping of interventions onto sub-processes in the RTR-model, the experimental setup implements interventions as perturbations in a single sub-process. It is assumed agents are homogenous, hence these perturbations will affect all agents equally and at the same time. The SIS mechanism adopted in our model is generally associated with disease spread, therefore we align our intervention logic to this setting. In a disease spreading setting, an intervention could be to provide antibiotics (this would reduce their viral load and thus make the population less infectious). Assuming this would reduce infectiousness by 20%, such an intervention would result in a reduction of radiation by 20% (α_*_ = *α* − 0.2). In line with this example, interventions are implemented as a system-wide reduction in a single sub-process (and corresponding parameter) halfway though the simulation (*t* = 25).

### Analysis strategy

Leveraging the experimental setup we will take the following steps during our analysis. First, to support the validity of decomposing the propagation mechanism, we will compare the behaviors of various decompositions in the RTR model without interventions. Second, to show how RTR behaves differently compared to traditional models, we will introduce interventions into the model. Third, to highlight the extent to which misspecification of the mechanism can have an impact on propagation dynamics under interventions, we will simulate interventions in a set of extreme scenarios. And lastly, to show the impact of the decomposition has on intervention strategies, we compare the effects of interventions of different sizes and at different targets.

Note that, due to the size of the experimental design, only a subset of results will be presented in the main text. We will use the synthetic network with random seeding and the scenario of an intervention in the Radiation process to illustrate our results throughout the main text. The remainder of the results, e.g. those for interventions in Transmission and Reception, and robustness checks with different seeding strategies, and the Facebook and AS-733 network structures, are included as part of the supplementary information (S5 File).

## Results

**Fig 2:**
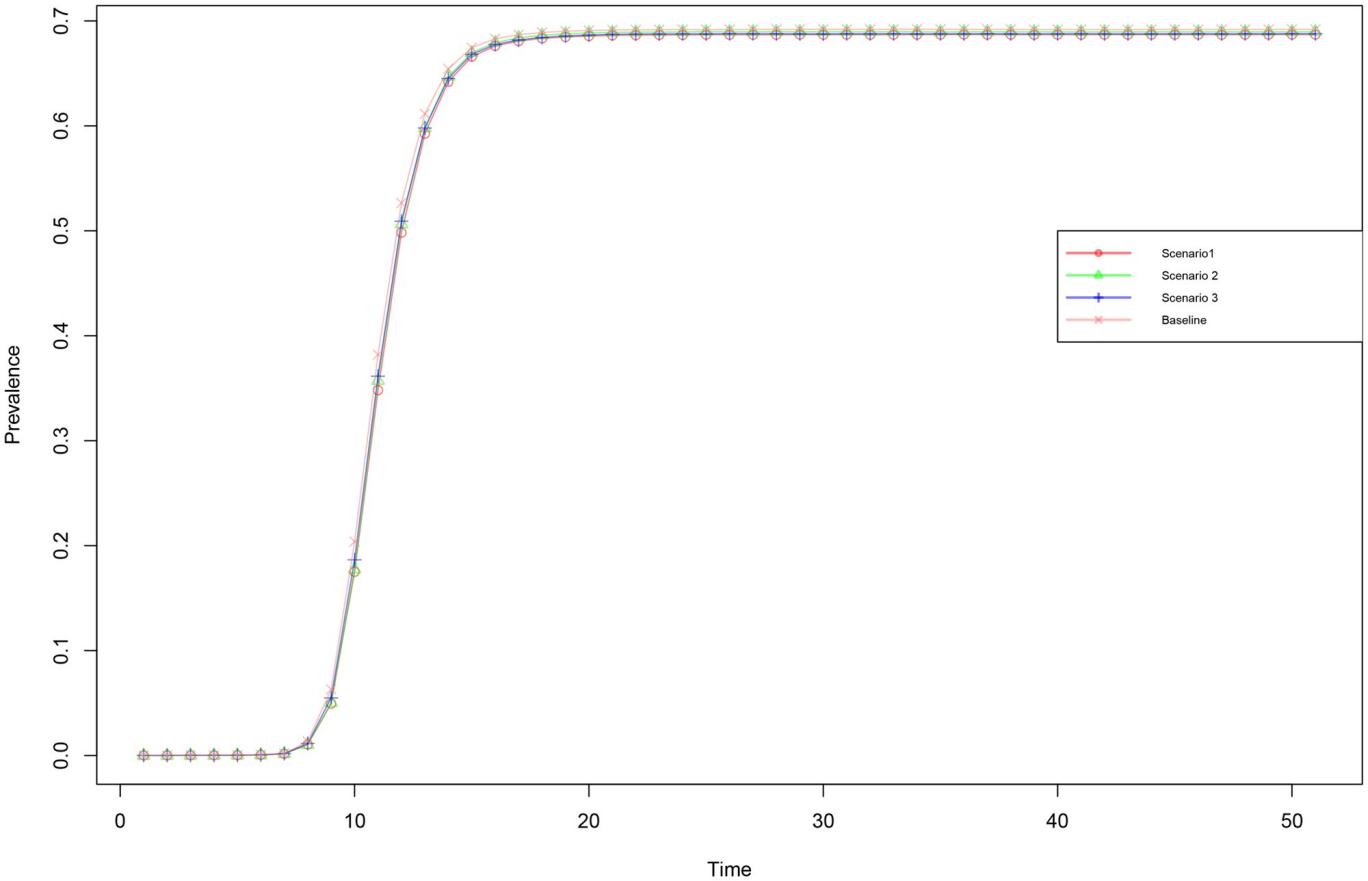
For network 1 with random seeding the mean prevalence over time is depicted for the four scenarios with the same overarching propagation likelihood (λ = 0.192). Scenario1, the red open circles, is a process with *α* = 0.4, *ϕ* = 0.6 and *η* = 0.8. Scenario2, the green triangles, a process with *α* = 0.6, *ϕ* = 0.8 and *η* = 0.4. Scenario3, blue pluses, a process with *α* = 0.8, *ϕ* = 0.4 and *η* = 0.6. Scenario 4, the pink crosses, a process with homogeneous sub-processes (*α* = *ϕ* = *η* = 0.5769).

The RTR framework, and the accompanying model, present a more detailed way of modeling propagation, as with any new model its behavior needs to be verified prior to being adopted in experiments. We do so by numerical comparing the prevalence numbers (% of the network affected) across the various decomposition scenarios (described in Table 2). The four scenarios are shown to achieve both propagation curves and dynamic equilibria levels that are graphically identical (Fig 2). A formal test of the resemblance of the equilibrium states reveals that the prevalence for each of these processes are statistically indistinguishable (*F* = 1.512 and *P* = 0.219), confirming that the differently decomposed scenarios are behaving identical. Note that our baseline scenario matches the traditional SIS model of propagation directly and behaves the same as all other decompositions, this indicates the SIS model is indeed a special case of the RTR model. This in turn validates the choice of using RTR implemented scenarios to compare to traditional SIS models.

**Fig 3:**
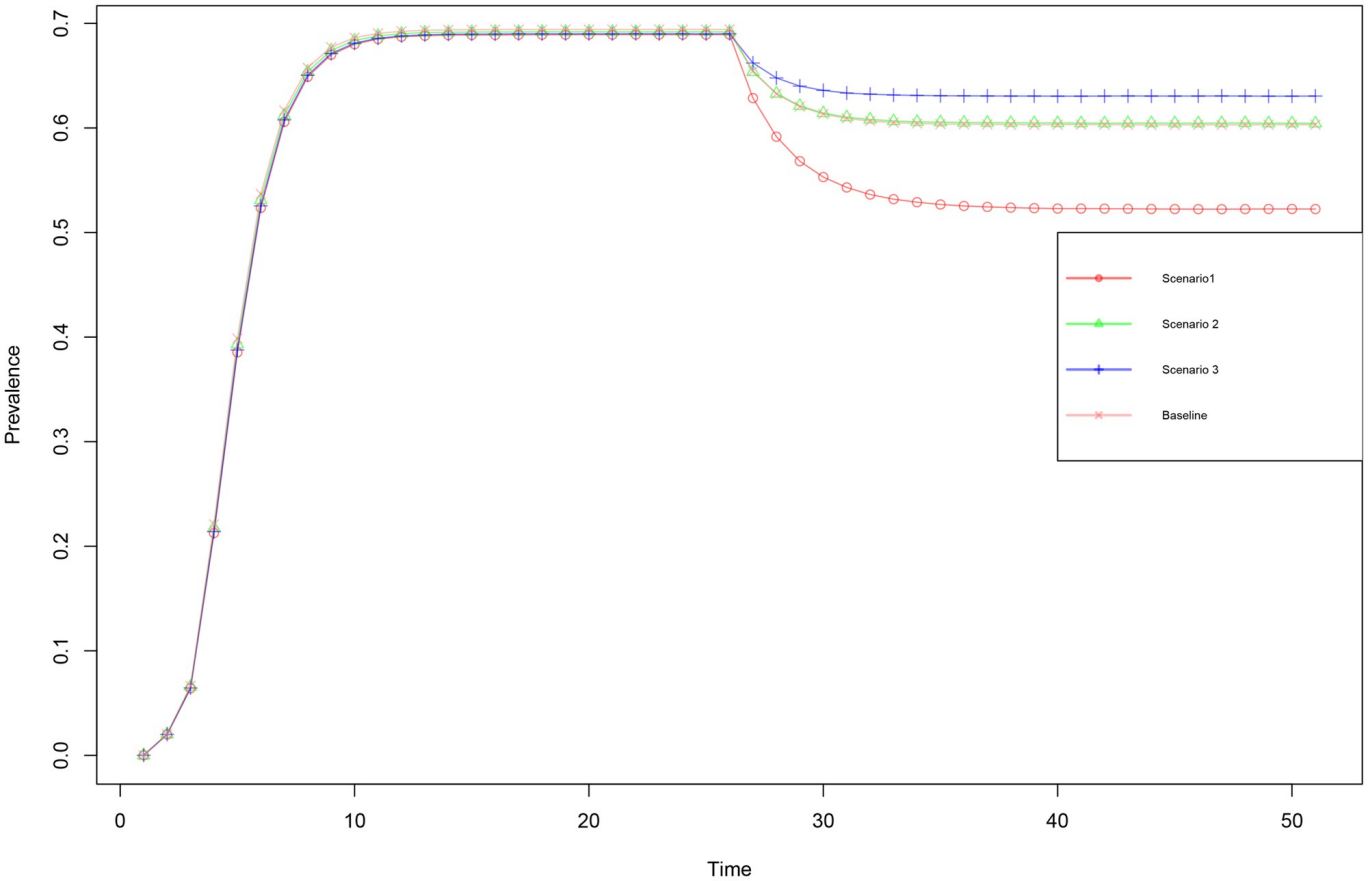
For network 1 with random seeding the mean prevalence over time is depicted for the four scenarios with the same overarching propagation likelihood (λ = 0.192). Scenario1, the red open circles, is a process with *α* = 0.4, *ϕ* = 0.6 and *η* = 0.8. Scenario2, the green triangles, a process with *α* = 0.6, *ϕ* = 0.8 and *η* = 0.4. Scenario3, blue pluses, a process with *α* = 0.8, *ϕ* = 0.4 and *η* = 0.6. Scenario 4, the pink crosses, a process with homogeneous sub-processes (*α* = *ϕ* = *η* = 0.5769). During the simulation at t = 25 an intervention (reduction of 0.2) in the **radiation** is applied.

Existing propagation literature suggests that, because of their similar composite propagation likelihoods, these scenarios will behave the same. This assumption is supported by results from the unperturbed setting, in which behavior is shown to be independent of decomposition. However, when perturbations are included in the model, this logic breaks down. We find that applying a single intervention (20% reduction in radiation) to the four scenarios results different prevalence curves (Fig 3). While the curves for the baseline and second scenario are very similar and lie nearly on top of one another, the first and thirds scenarios show clearly identifiable differential behavior. Table 3, presenting the same results in numeric terms, more clearly shows the effective reduction of prevalence obtained by intervening for each of the scenarios. These findings indicate that scenarios do behave differently when perturbed, and that for intervention purposes these scenarios are fundamentally different. As scenarios differ only in decomposition, it becomes evident that the decomposition is the driver of these differences and what determines the effectiveness of interventions in propagation.

**Table 3:** Intervention effects for four scenarios, under random seeding on network 1.

Scenario	Pre-intervention prevalence	Post-intervention prevalence	Intervention effect (relative effect)	effect relative to baseline	Composite λ post intervention
**Baseline:** *(Rad = Tra = Rec = 0.5769)*	0.697	0.603	-0.091 (-13.1%)	100.0%	0.125
**Scenario 1** *(Rad = 0.4 Tra = 0.6 Rec = 0.8)*	0.697	0.541	-0.156 (-22.4%)	176.0%	0.096
**Scenario 2** *(Rad = 0.6 Tra = 0.8 Rec = 0.4)*	0.697	0.613	-0.083 (-12.0%)	94.3%	0.128
**Scenario 3** *(Rad = 0.8 Tra = 0.4 Rec = 0.6)*	0.697	0.640	-0.057 (-8.4%)	64.4%	0.144

This table shows for each scenario the relative effects of an intervention (-0.2 reduction) in radiation

Note that while only a subset of results are presented in the main manuscript, these results to be robust across intervention targets, network structures and seeding strategies. The radiation intervention results are consistent with those from Transmission and Reception (see Figs A and B, in S4 File). Similarly, (S5 File) shows that that the seeding strategy has no impact on our results, and we find that while different overall prevalence levels across network structures (and thereby different intervention effects in an absolute sense) occurs, the relative impact of the decomposition (the extent that different decomposition scenarios differ from the baseline scenario) remains constant across networks.

Our intervention results highlight significant variation in intervention effectiveness stemming from variations in the decomposition of the propagation mechanism. This should not come as a surprise when one considers the decomposition of the propagation rate (λ = *α* × *η* × *ϕ*). From the decomposition itself we can deduce that the net effect of an intervention (an absolute change in one of the parameters of the sub-processes) on the composite propagation rate (λ) will be a multiplication of the intervention size and parameters for the remaining sub-processes. While this net effect determines the observed intervention effectiveness in our simulations, it becomes evident that the effective reduction of λ achieved by an intervention is conditional upon the decomposition. Therefore, only when we know the decomposition we can make informed decisions regarding the effects of an intervention strategy.

**Fig 4:**
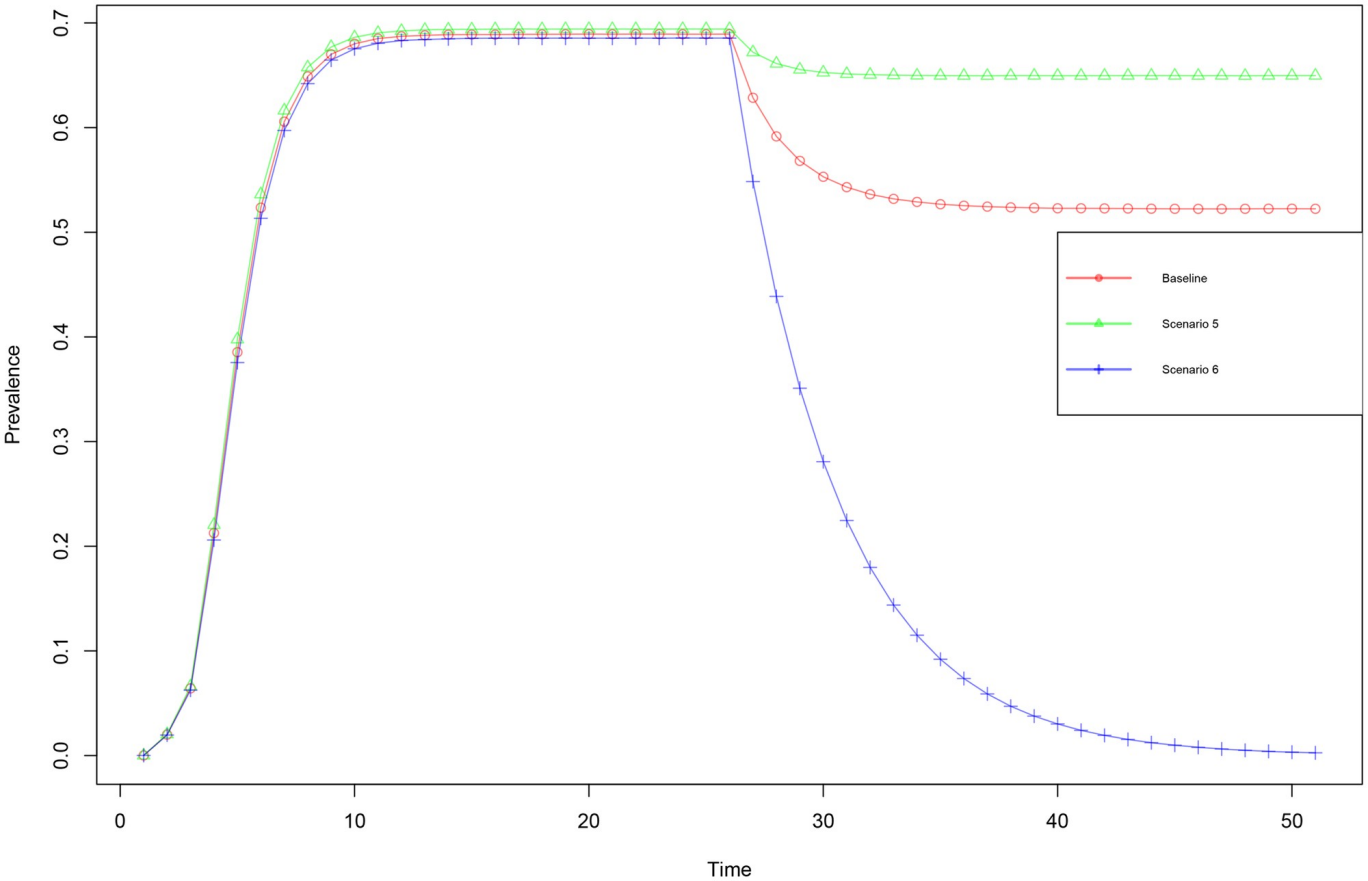
For network 1 with random seeding the mean prevalence over time is depicted under random seeding on network 1 for the 3 scenarios with the same overarching propagation likelihood (λ = 0.192). The baseline scenario, the red open circles, a process with homogeneous sub-processes (*α* = *ϕ* = *η* = 0.5769). Scenario 5, the green triangles, a process with *α* = 1.0, *ϕ* = 1.0 and *η* = 0.192. Scenario6, blue pluses, a process with *α* = 0.2, *ϕ* = 1.00 and *η* = 0.96. During the simulation at t = 25 an intervention (reduction of 0.2) in the radiation is applied.

To further explore the effect of incorrect specification, we extend the radiation intervention setting (in network 1 with random seeding) with two additional scenarios to investigate how large the variation in effectiveness of a given intervention can be when the mis-specification of the propagation process is substantial. We generated two additional scenarios that keep the composite propagation likelihood the same (λ = 0.192), but differ in their resilience to the proposed intervention. Building on the notion of that the net effect on the composite λ drives the intervention effectiveness the first additional scenario (scenario 5) we will maximize the radiation parameter pre-intervention (*α* = 1.0, *ϕ* = 1.0 and *η* = 0.192). We assume this to be the most resilient scenario as the intervention has the smallest impact relative to the size of the radiation parameter. In the second additional scenario (scenario 6) the radiation parameter is set equal to the intervention size (*α* = 0.2, *ϕ* = 1.0 and *η* = 0.96), this will be the least resilient scenario as the intervention effectively reduces the radiation to 0. The results of simulations with these additional scenarios (Fig 4, Table 4) show that the effects of a given intervention can differ more than an order in magnitude for a given intervention, indicating just how important the correct specification is.

**Table 4:** Intervention effects for two additional extreme scenarios, under random seeding on network 1.

Scenario	Pre-intervention prevalence	Post-intervention prevalence	Intervention effect (relative effect)	effect relative to baseline	Composite λ post intervention
**Baseline:** *(Rad = Tra = Rec = 0.5769)*	0.694	0.603	-0.091 (-13.1%)	100.0%	0.125
**Scenario 5** *(Rad = 1.0 Tra = 1.0 Rec = 0.192)*	0.694	0.642	-0.045 (-6.4%)	48.8%	0.1536
**Scenario 6** *(Rad = 0.2 Tra = 1.0 Rec = 0.96)*	0.686	0.032	-0.682 (-99.5%)	748.2%	0.0

The extreme scenarios again have the same overarching propagation probability (λ = *α* × *η* × *ϕ* = 0.192), but are decomposed differently. Scenario 5, the most resilient scenario, has a decomposition of [*α* = 0.192, *ϕ* = 1.0 and *η* = 1.0], whereas scenario 6, the most fragile scenario, has a decomposition of [*α* = 0.2, *ϕ* = 0.96 and *η* = 0.1]

**Fig 5:**
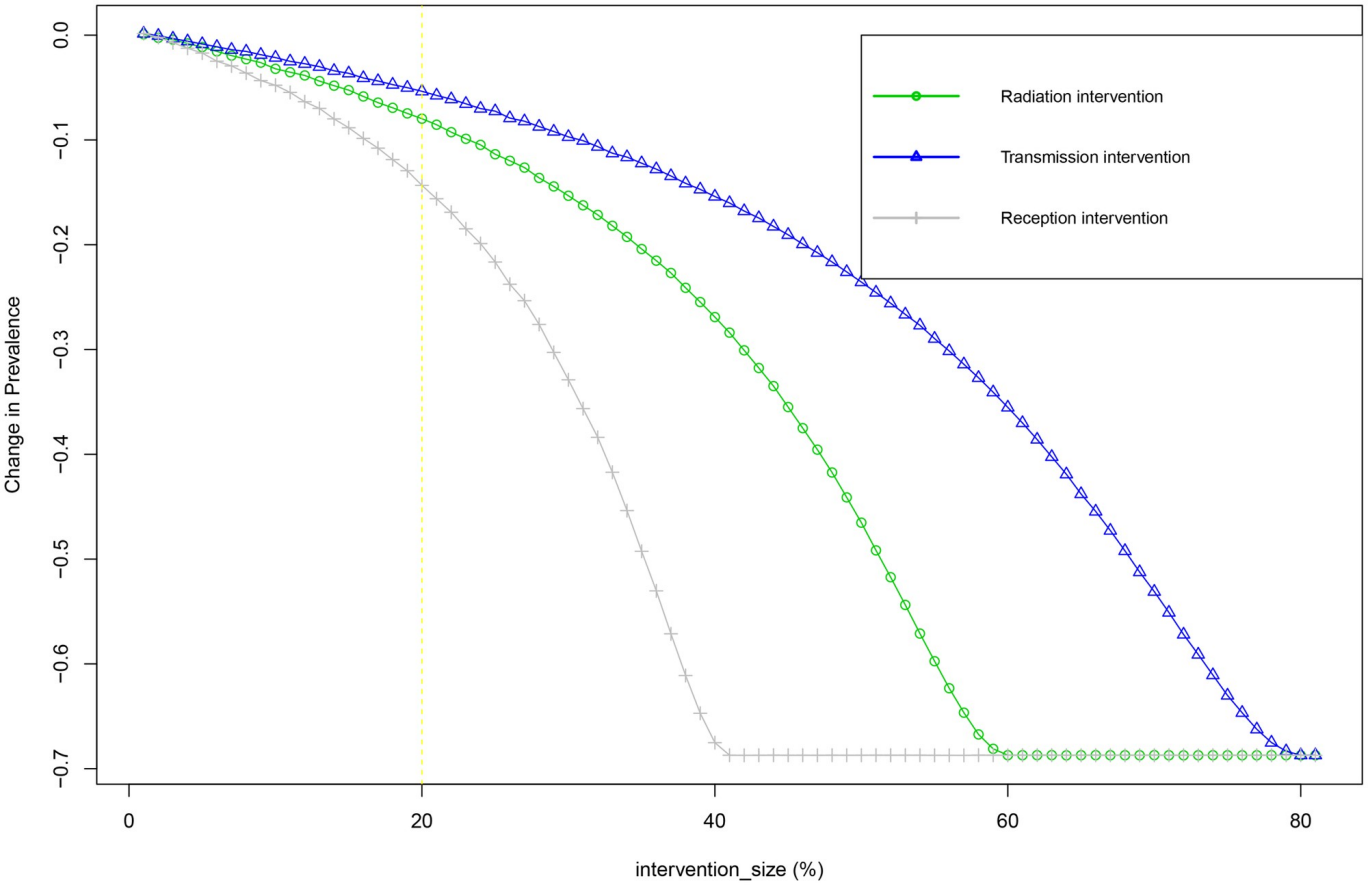
The mean reduction in prevalence (y-axis) in scenario 3 (*α* = 0.8, *ϕ* = 0.4 and *η* = 0.6) for interventions of various size(x-axis) across the different target processes, green circles for interventions in Radiation (*α*), blue triangles for interventions in transmission (*ϕ*), and gray pluses for interventions in reception (*η*).

Thus far, the presented results have assumed intervention size to be fixed, and while this has been useful exercise for showing the relevance of correct specification, it might not be the most realistic representation of interventions in practical settings. In practice a policy maker faces a given propagation phenomenon, and thus a given mechanism of propagation, for which the policy maker wants to intervene most effectively. As an illustration, assume a policy maker is faced with a phenomenon that is accurately described by scenario3 (for similar analysis for the other scenarios see Figs A and B in S6 File) one wants to know the potential impact of a given intervention, or how much one should intervene to obtain the desired impact. So rather than assuming an intervention of fixed size the assumption is that intervention sizes can vary. To incorporate this in our model we allow intervention sizes to span the full range (0% to 100%, with increments of 1%) for all three targetable sub-processes and explore the impact. Similar to the results presented in the previous tables, for each such intervention we calculate the effect on the prevalence (unperturbed prevalence—prevalence after intervention), and plot these values. The results (Fig 5) indicate three things. First, in line with previous observations, it shows that for an intervention of a given size (i.e. the vertical yellow dashed line corresponds with the previously discussed intervention of size 0.2), the effectiveness of that interventions varies strongly depending on which sub-process is targeted. Second, it shows that for a given mechanism there seems to be a single most effective target for intervention. For this specific scenario, the interventions in the transmission sub-process are particularly impactful. Comparing these results across various mechanisms (S6 File), however reveals that the best targeted sub-process varies across scenarios, and that the most effective target for interventions (Radiation, Transmission or Reception) depends on the decomposition of the propagation mechanism. More specifically, we find that the most effective target for intervention is the sub-process that has the smallest parameter value. This is somewhat counter intuitive, as this means that the most noticeable sub-process (the one that has the highest likelihood of being observed) is in fact the least efficient target of intervention. Third, we observe a non-linear effect of intervention size, meaning each additional 1% that can be added to size of the intervention becomes increasingly effective (up until the point where that sub-process is effectively eradicated).

## Discussion

Modeling the propagation mechanism through our Radiation-Transmission-Reception decomposition not only brings more conceptual clarity to the propagation process, but the results presented in the previous section also show that such decomposition yields important empirical insights that cannot be gained using a standard single parameter (λ) model.

Our results (Fig 3 / Table 3) show that the effectiveness of interventions, which conceptually map onto changes in sub-processes, depend greatly on how the underlying propagation process is decomposed; even with composite rate of propagation (λ) constant, one single intervention can have effects that differ two orders of magnitude depending on the values for Radiation, Transmission and Reception (see Fig 4 / Table 4), and that intervention effectiveness can differ roughly by a factor eight from the traditional single parameter models in extreme settings. The findings indicate that without decomposition of the mechanism of propagation, substantial over- or underestimation of intervention effectiveness can occur.

Our findings highlight the responsibility of researchers to accurately adjust the model to a given context, and their obligation to be critical when generalizing results from other settings. Clearly specifying a model in RTR terms can be helpful for both these aspects, and has the added benefit of informing one’s potential intervention strategies. For instance, an influenza epidemic where spread is driven primarily by high radiation [51] will have different intervention effectiveness profile (in terms of Fig 5) than for instance the propagation of financial distress among firms in which the reception component is strong [67].

Even within a specific context, competing conceptual interpretations of the same phenomenon exist. For instance, the propagation of information on social media has been modeled using an emphasis on radiation [46, 68], an emphasis on transmission [47] and an emphasis on reception [4]. Similarly, the propagation of epidemics has been modeled using an emphasis on radiation [51], an emphasis on reception [44, 52] and an emphasis on transmission and reception [36]. Only by adopting a framework that encompasses all these models we can start translating their outcomes and move the field of propagation forward.

Note that by highlighting these differences we do not imply that any of these authors are wrong in their specific context or their modeling choices. Rather, our claim is that there are implicit modeling assumptions being made about the nature of the propagation mechanism that could greatly impact the generalizability of the respective results. This in turn leads to conflicting results about the effectiveness of interventions, which negatively impacts our chances to build a cumulative body of knowledge.

### Practical implications

From a practical point of view, the observed potential impact of mis-specification is undesirable. In practical settings such variance in the effectiveness is even more worrisome as designing and implementing network intervention is too costly an endeavor to follow a shotgun approach. The RTR model allows for a more targeted approach where the intervention can be fine-tuned to the details of how the underlying propagation mechanism works in that particular context. What is more, the RTR model helps researchers make these mechanisms explicit, which will enable improved targeting of intervention, and facilitates communication of results across the wide variety of scientific disciplines that deal with propagation. This in turn will translate into a more generalizable understanding of propagation dynamics and interventions, which can consequently be used by practitioners in different fields that aim to design and implement effective interventions.

Various settings can be mentioned that could leverage this approach. For example in the context of HIV prevention, understanding the mechanism responsible for HIV transmission allows one to better target interventions strategies. In this specific setting various interventions strategies are available; Anti-retro-viral drug treatment (ART) allow for viral suppression and thus can lower radiation, Transmission can be reduced by safer sex and thus can be affected by making sure condoms are being used, and recently developed Pre-exposure prophylaxis (PrEP) allows for greatly reducing susceptibility to HIV and thus affects reception. While each of these interventions is effective from a clinical standpoint and can reduce HIV incidence, understanding the how to implement such intervention effectively requires a detailed understanding of the propagation mechanism.

Similarly in the context of marketing, being able to differentiate between the effectiveness of strategies aimed at increasing the sharing of information regarding a product (Radiation), the effects of different media used to send an advertisement (Transmission), and those that affect the willingness to adopt (Reception) is crucial. Such insight can only obtained when a model is used that allows for decomposing the propagation mechanism.

In the setting where more complex information needs to be exchanged, such as knowledge transfer about best practices between business units in a multinational organization, the RTR model can help to decide on the right intervention(s). For instance, [31] showed the difficulty of knowledge transfer to depend, among other things, on the relationship between the knowledge source and knowledge recipient (hinting at the relevance of the Transmission sub-process) and the absorptive capacity of the knowledge recipient (hinting at the Reception sub-process). Moreover, [31] investigated the effectiveness of a number of knowledge transfer methods (i.e. radiation mechanisms) and found that some methods such as best practice manuals, newsletters and company video films are mostly effective in the initial phases of knowledge transfer. Conversely, other methods such as personnel rotation and physical visits from the knowledge source to the knowledge recipient (emphasizing Transmission and Reception) were mostly effective during the later phases of knowledge transfer. The RTR model provides a framework for studying such complex knowledge transfer methods by linking the methods to specific sub-processes, which in turn allows such lessons to be adopted in other domains.

### Limitations and future research

The simulation methodology adopted in this paper has its limitations, while we designed our mechanism of propagation to fit our goals of showing the potential impact of decomposing the propagation process, applying our model to specific context will require substantial effort. As understanding the details of the propagation mechanism is likely hard, and our model is more detailed in describing such mechanism, the need expert domain knowledge, or data, is substantially increased, which puts additional constraints on the requirements for effective modeling efforts.

Furthermore, the applied model makes some simplifying assumptions which need to be further explored. For example it is currently assumed that all actors are homogeneous, and thus behave the same. While such assumptions reduce the noise in the results, it is known that in most real world systems actors in fact do differ in terms of their contagiousness, transmission medium, and susceptibility, making the inclusion of heterogeneity crucial extension to our model. Similarly, the effects of network structure and network seeding as a key drivers of propagation dynamics remains somewhat under explored. Exploring how the network structure and the mechanism of propagation interact and have a combined effect on the propagation dynamics, and exploring the interactions between seeding strategies and the mechanism will be fruitful avenues of future research.

## Supporting information

S1 File Overview of symbols used in the manuscript and their meaning. (PDF)

S2 File Examples of various intervention strategies in practice. (PDF)

S3 File Translation from RTR to traditional propagation models. (PDF)

S4 File Intervention effects for interventions in Transmission and Reception. (PDF)

S5 File Checking the robustness of the simulation results. (PDF)

S6 File Intervention effects for interventions in scenario 1 and 2. (PDF)

S7 File All data and scripts associated with the analysis in this manuscript. (ZIP)
