# Supplementary material for: The Radiation-Transmission-Reception (RTR) model of propagation: Implications for the effectiveness of network interventions
Source: PLoS One. 2018 Dec 5;13(12):e0207865. doi: 10.1371/journal.pone.0207865 (PMC6281238; doi:10.1371/journal.pone.0207865)
Supplement: S1 File — (PDF) [file pone.0207865.s001.pdf]

This table provides an overview of all the symbols used in the equation in this manuscript, and describes their meaning.

Symbol	Meaning
$s_{i,t}$	The state of actor i at time t.
$A_{i,t}$ ($\alpha_{i,e,t} \in A_{i,t}$)	A vector capturing the characteristics which influence the radiation per outgoing edge e at time t.
T^{rad}	The radiation memory duration, i.e. the number of time units after a change of state that this change can cause radiation.
τ_{rad}	The memory inflation factor, i.e. the extent to which past changes are amplified or dampened.
u	The threshold for radiation, i.e. the amount of change in state required to result in radiation.
$p^{out}_{i,e,t} \in P^{out}_{i,t}$	The pulse being radiated from actor i to edge e at time t.
T^{tra}	The memory duration for transmission sub-process, i.e. the number of time units a radiated pulse can cause transmission.
τ_{tra}	The memory inflation factor for the transmission sub-process, i.e. the extent to which past pulses are amplified or dampened during transmission.
$\phi_{e,t}$	A vector of edge characteristics which influence transmission.
$p^{in}_{e,j,t}$	The pulse receive by actor j from edge e at time t.
T^{rec}	The memory duration for the reception sub-process, i.e. the number of time units a transmitted pulse can cause reception.
τ_{rec}	The memory inflation factor for reception sub-process, i.e. the extent to which past pulses are amplified or dampened during reception.
$\Psi_{i,t}$ ($\eta_{e,i,t} \in \Psi_{i,t}$)	A vector capturing actor specific characteristics influencing the reception per incoming edge e on time t.
q	The threshold for the reception sub-process, i.e. the amount of incoming pulses required to result in reception.
