# Supplementary material for: The Radiation-Transmission-Reception (RTR) model of propagation: Implications for the effectiveness of network interventions
Source: PLoS One. 2018 Dec 5;13(12):e0207865. doi: 10.1371/journal.pone.0207865 (PMC6281238; doi:10.1371/journal.pone.0207865)
Supplement: S2 File — (PDF) [file pone.0207865.s002.pdf]

Examples of interventions in propagation in practical settings. For each setting it shows how the propagation process can be divided into three sub-processes and how different interventions are linked to specific sub-processes.

Setting	**Radiation**		**Transmission**		**Reception**	
	Process	Intervention	Process	Intervention	Process	Intervention
**Spread of financial crisis**	A bank becomes insolvent and defaults on a loan	*Capital injections* from central banks may change the state of the insolvent bank, making it solvent again before any propagation can occur. This prevents the radiation of a signal.	The loans between banks causes the default of one bank to result in a loss for a connected bank.	*Guarantees, securities and derivatives* can ensure defaulted loans are still (partially) covered, reducing the signal which reaches the alter.	The other bank will incur a loss from the defaulted loan and can absorb this loss or change state to become insolvent.	*Enforcing an increase in the liquidity buffers* results in banks being less likely to also become insolvent when incurring losses from other defaults.
**Contagion of disease**	A person becomes sick and spreads the viral load/germs in the direction of others, i.e. through sneezing.	*Quarantine interventions*, such as telling people to stay at home, reduce their interaction with friends and hence reduce the chance of spreading the disease to these friends.	The transfer of viral load/germs to other people (alters), i.e. being hit by a sneeze or touching a contaminated surface.	Getting those people who are infected to *wear protective masks, and/or wash their hands* extensively will reduce the viral load/germs an individual will spread and hence reduce the transmission of the virus to other people.	The alters respond to the viral load/germs being received. They attempt to fight off the infection, and either succeeds, so the disease does not propagate, or fail and gets sick.	*Immunization* decreases the susceptibility of actors, as immunized actors are considered to react differently to incoming signals. Through immunization, the reception process of these actors is changed.
**Adoption of innovation**	A firm adopts a new production technique and its employees start to talk about this adoption.	Communication or visibility is reduced or increased by means of *confidentiality or advertising agreements.*	Other firms becoming aware of the adoption, through interaction. This can be by communication or observation.	By *using a different means of communication or changing the transparency* the extent to which the signal reaches an alter can be changed.	Other firms make a decision on whether to also adopt the innovation, based on the signals they receive via their network.	By *changing the investment costs* (subsidizing or licensing) the other firms will become more, or less, prone to adopt the innovation.
**Diffusion of information or knowledge**	A person has a certain piece of novel information and decides to share this.	*Rewarding sharing activity with status* (as is often done on online forums) incentivizes the sharing of information.	Communication among individuals allows the information to be spread from one person to another.	By *using a different medium* (e.g., telegram vs. videoconferencing) the richness of communication can be changed, thus improving or reducing the extent to which the information is understood during transmission.	The alter receives the information and fits this to his or her existing knowledge and decides to accept or reject the information, in full or in part.	By *decreasing or increasing the utility* (novelty) of the information being shared, the willingness to accept such information can be increased.
