# Supplementary material for: The Radiation-Transmission-Reception (RTR) model of propagation: Implications for the effectiveness of network interventions
Source: PLoS One. 2018 Dec 5;13(12):e0207865. doi: 10.1371/journal.pone.0207865 (PMC6281238; doi:10.1371/journal.pone.0207865)
Supplement: S3 File — (PDF) [file pone.0207865.s003.pdf]

The definition of propagation allows it to cover processes in a wide range of fields and disciplines. As many discipline have converged into their own standards and sets of rules for modeling the specific propagation process there exists a forest of different propagation models in literature. In general four basic classes of models can be identified; SIS/SIR models [1], Bass(-like) models [2], threshold models [3, 4] and cascade models [5].

SIS/SIR models

By far the largest class of models of propagation are based on SIS/SIR type of models. These model stem from the field of epidemics and owe their name to the different compartment, or states, an actors in the system can be in. Actors are either **S**usceptible, **I**nfected or **R**emoved, hence resulting name SIS/SIR model. In this type of model each change of compartment is given by a rate, the process by which actors change from Susceptible to Infected is given by means of an infection rate (λ), and a process by which actors change from Infected to Susceptible/Removed by the recovery rate (ρ), or death rate (γ), these processes are considered to be stochastic and dependent on the interactions among actors. Even though many extensions of the traditional SIR/SIS models exist (most of them add extra state to the model, for example by allowing actors the be exposed, or temporary immune) for convenience the simple SIS model will be elaborated in order to introduce the logic of tis class of propagation model.

The population in this type of model is divided in different compartments of actors, each with a different state. These compartments interact at a certain rate which is based on the size of the population in each of the available compartments. By considering the average rates by which the actors change from one state to another, this models allow writing the dynamics of propagation as a set of differential equations.

Assume a set of N actors which are either susceptible (S) or infected (I) such that $N = S + I$. In this case the SIS model is described by:

$$\frac{\Delta I}{\Delta t} = -\rho I + \frac{\beta S I}{N} \tag{1}$$

$$\frac{\Delta S}{\Delta t} = -\frac{\beta S I}{N} + \rho I \tag{2}$$

In which β is the rate of interaction.

This model assumes random interactions among actors, essentially considering a scenario with homogenous mixing. More complicated extensions have been developed which include the notion that there is a network underlying the propagation process. The network causes actors to have a certain amount of connections, which affects their ability to both infect others, and be infect by others, resulting in the following formulation [6]:

$$\frac{\Delta I}{\Delta t} = -\rho I + I\langle k\rangle\lambda(N - I) \tag{3}$$

$$\frac{\Delta S}{\Delta t} = \rho I - I\langle k\rangle\lambda(N - I) \tag{4}$$

In which $\langle k\rangle$ is the average number of edges (connections) per vertex (actor).

Even more complex variations of this model can also capture heterogeneity in the network structure [6, 7], but the logic of the model remains the same throughout. These models leverage the assignment of actors to groups, which are called compartments, based on their state and number of connections. It is assumed that all actors in the same compartment behave similarly. Consequently these models are called mean-field models. Mean-field models can be roughly divided in three camps [8]:

- Individual-based mean-field approach (IBMF): In which it is assumed that every actor belongs to compartment of the system with a certain probability, and that all actors within a compartment behave similarly.
- Degree-based mean-field approach (DBMF): Here compartments are based on the number of ties an actor has. It is assumed that every actor with the same number of ties, its degree, behaves statistically similar.
- Generating function approach: A special case method for scenarios in which infected actors are removed after infection. Which builds on the notion that the likelihood of finding a tie is related to the probability of transmission of the disease, which is constant for the complete population.

[8] provide an mathematical representation of these models and an extensive overview of their characteristics and differences. Each of these approaches leverages the same logic, it considers the average propagation behavior per compartment in the system, by

going over the dynamics of each group one will get the dynamics of the system as a whole. The main argument for doing so is that this allows these models to step away from the apparent chaotic behavior of the individual actor level [9]. Also combining set of actors, significantly reduces the complexity of the formulation of the propagation behavior. The mean-field approach further enable using an analytical methodology to untangle the propagation dynamics, which in turn has resulted in closed form solutions to many propagation problems [8].

Cascade models

A second class of propagation models is cascade models (e.g. [10]). Similar to the SIS models this type of model considers a stochastic process in which an 'infected' actor will propagate its behavior towards it connected neighbors. Potentially resulting in the occurrence of cascades of such behavior. An important characteristic of cascade models is that they are build on the notion of momentum. This means that once an actor changes state, it will sent out a signal towards a neighbor only once! Regardless of the outcomes of this step it loses its momentum after this initial shock and will become inactive thereafter.

As [5] puts it '*The basic cascade model therefore can be described by a process which starts with an initial set of active* I_0 *actors. The propagation process unfolds in discrete steps according to the following randomized rule. When node i first becomes active in time step* t*, it is given a single chance to activate each currently inactive neighbor* j*; it succeeds with a probability* $p_{i,j}$ *—a system-wide parameter— independently of the history thus far*'.

Threshold models

The third class of propagation models, threshold models (e.g. [4]) assume a different type of propagation mechanism. These models assume that adoption of a certain state is a consequence of the states of its connected neighbors. In each time step all actors will therefore reconsider their state. Each actor will look at its direct neighbors and change state if a large enough proportion of the neighbors has adopted an alternative state. Whether the proportion of the neighbors is big enough to change state, depends on the adoption threshold of the focal actor.

[11] describe the most basic threshold model, the linear threshold model, in which it is assumed that each actor has an individual threshold for changing its behavior. This means that for each actor the proportion of neighbors needed differs, they al have a different randomly chosen threshold. This model consists of two elements:

- A set of edges E with a positive weight w_{ij} on each edge from i to j, which indicates the influence of actor i on j . It is dictated that $\sum_{w \in N(j)} w_{ij} \leq 1$, where $N(j)$ is the set of nodes with edges to j.
- A set of thresholds Θ, containing a threshold θ_i for each node i. θ_i is chosen uniformly at random from $[0, 1]$

Due to some external event a set of nodes will initially adopt the alternate behavior, these nodes are claimed to be active. At any discrete time step $t = 1, 2, 3, \ldots$ any inactive node j becomes active if the fraction of active neighbors exceeds its threshold:

$$\sum_{A \in N(j)} w_{ij} \geq \theta_j \tag{5}$$

Where A is the set of active nodes. While far more complicated threshold models can be considered, an indeed models considering also the absolute number of neighbors [11, 12] are quite common, all threshold models follow this fundamental logic of updating the state of actors based on the state of the connected neighbors.

Bass-like models

The fourth class of models are the Bass-like models. The models often used in the field of marketing derive their name from the traditional Bass model [2] which was used to describe the adoption of innovation among actors. It consists of a differential equation describing the process of how such adoption occurs. The basic premise of the model is that adoption is driven by two forces, random adoptions, and influence from other actors. Each actor is classified as innovator or as imitator. Innovators are those which have a high probability to adopt a innovation at random, and imitators are those which are more likely to adopt due to influence. The speed and timing of adoption depends on their degree of innovativeness and the degree of imitation among adopters.

The basic Bass model is formulated as:

$$\frac{f(t)}{1-F(t)} = p + qF(t) \quad (6)$$

In which $f(t)$ is the change in the proportion of adopters, $F(t)$ is the proportion of adopters, p is the rate of innovation (random adoption) and q is the rate of imitation.

It should be noted that the Bass model does not mention a structure of interactions, and hence this model ignores the impact of the network structure. It effectively assumes that actors have full information on the state (changes) of actors in the system, which can be translated into assuming a completely connected network, in which each actor is connected to each other actor.

The generic RTR-model

Equations 1, 2 and 3 (in the main manuscript) describe the RTR-model in its most generic form. Note that this model is far more complex than traditional models and as such allows for capturing mechanisms of propagation, that include threshold effects, heterogeneous actors, temporal effects and complex interactions among sub-processes. In comparing the RTR model with traditional models which assume far less complex mechanisms, such model complexity is often unnecessary, and for that reason the general RTR model can be tuned down to describe simpler mechanism. In the following sections we will do so while relating the RTR model to traditional models.

The binary state RTR-model

By making additional assumptions the complexity of the generic RTR-model can significantly be reduced. The generic RTR-model describes the state of vertex as a continuous variable while many studies have assumed a propagation processes in which the state of a vertex can be described by a binary variable in which actors can have one of two states ($s_{i,t} \in \{0,1\}$). For example when considering the spread of disease in which actors are either sick or not, an actor adopts an innovation or certain behavior or it does not. A binary state version of the RTR-model can be easily obtained by adjusting the reception sub-process and incorporating the notion that any change in state will be of size 1, and that once actors are in state 1 their state cannot further increase their state. The radiation and reception functions in binary state model can

consequently be rewritten. For Radiation we get:

$$\sum_{t'=t-T+1}^{t} \sum (A_{i,t'} \times P_{i,t'}^{out} \times (\tau_{rad})^{(t-t')+1}) = \begin{cases} 1 & \text{if} \quad \Delta s_{i,t} \geq u \\ 0 & \text{if} \quad \Delta s_{i,t} < u \end{cases} \tag{7}$$

In which u once again is the radiation threshold.

Similarly the reception sub-process can be formulated as:

$$\Delta s_{i,t} = \begin{cases} 1 & \text{if} \quad s_{j,t} = 0 \quad \text{and} \sum_{t'=t-T_{rec}+1}^{t} \sum (P_{j,t'}^{in} \times \Psi_{j,t'} \times (\tau_{rec})^{(t-t')+1}) \geq q \\ 0 & \text{if} \quad s_{j,t} = 0 \quad \text{and} \sum_{t'=t-T_{rec}+1}^{t} \sum (P_{j,t'}^{in} \times \Psi_{j,t'} \times (\tau_{rec})^{(t-t')+1}) < q \\ 0 & \text{if} \quad s_{j,t} = 1 \end{cases} \tag{8}$$

As the transmission function does not take into account the state of the vertices it will remain as described in the generic RTR-model.

Clearly, studying scenarios in which the only potential change in state does not pass the radiation threshold ($\Delta s_{i,t} = 1 < u$) is trivial as they will never result in propagation. However, to make sure the model mutually exclusive and collectively exhaustive this option is not to be excluded from the model description.

The stochastic binary state RTR-model

Taking a closer look at the propagation literature indicates that all commonly adopted models of propagation consider the propagation process to be stochastic rather than deterministic, as has been done so far when describing the RTR-model. The SIS/SIR models, commonly studied in epidemiology and underlying a large part of the propagation literature, considers a stochastic process and binary states. While the binary state RTR-model previously introduced is deterministic, this version of the model can easily be converted into a stochastic version very similar to the SIS/SIR models.

The binary nature of the state of the vertices makes it trivial to consider the size of a change in state or the size of the signal. What matters is a whether there is a signal or not. From this idea, the step towards considering the probability that such a signal is present is relatively small. Such a switch would effectively change the emphasis of the

model from size of the signal (read deterministic) towards the probability that a signal occurs (read stochastic).

In a stochastic version of the RTR-model the radiation likelihood describes the probability that a change in state (which can only be of size 1) will be larger than the radiation threshold, and thus yields any signal towards the outgoing edge(s). This probability is only influenced by a function of the radiation properties $(A^*_{i,t} = f(A_{i,t}) = (\alpha^*_{i,e,t})|e \in E^{out}_{i,t} = f(A_{i,t}))$ of the actor sending the signal(i) and the memory in the system. Therefore it can be formulated as:

$$p^{out}_{i,e,t} \sim Bern(p) \quad \text{in which} \quad p = 1 - \prod_{t'=t-T_{rad}+1}^{t} (1 - (\Delta s_{i,t'} \times \alpha^*_{i,e,t'} \times (\tau_{rad})^{(t-t')+1}) \tag{9}$$

When the propagation process is assumed to be stochastic the transmission sub-process describes the chance that a radiated signal is transmitted over an edge. This chance depends on the presence of an incoming signal, a function of the edge specific characteristic $(\phi^*_{e,t} = g(\phi_{e,t}))$ and the memory in the transmission. It can therefore be formulated as:

$$p^{in}_{e,j,t} \sim Bern(p) \quad \text{in which} \quad p = 1 - \prod_{(t'=t-T_{tra}+1)}^{t} (1 - (\phi^*_{e,t'} \times p^{out}_{i,e,t'} \times (\tau_{tra})^{(t-t')+1})) \tag{10}$$

The reception sub-process in a stochastic propagation scenario refers to the chance that the sum of the incoming signals aggregates into a signal that in fact surpasses the reception threshold. It will consequently can result in a state change of the receiving actor. This clearly depends on the state of the receiving actor (as it need to be able to change state) and a function of the reception parameters $(\Psi^*_{j,t} = h(\Psi_{j,t'}) = (\eta^*_{e,j,t})|e \in E^{in}_{j,t}))$ and the memory in the reception sub-process. Rewriting the binary reception formulation (equation 7) into a stochastic version yields:

$$\Delta s_{j,t} = \begin{cases} \sim Bern(p) \quad \text{if} \quad s_{j,t} = 0 \\ 0 \quad \text{if} \quad s_{j,t} = 1 \end{cases} \tag{11}$$

In which $p = 1 - \prod_{t'=t-T_{rec}+1}^{t} \prod (1 - (P^{in}_{j,t'} \times \Psi^*_{j,t'} \times (\tau_{rec})^{(t-t')+1}))$.
The SIS/SIR models can be denoted as a special case of this general stochastic form with two addition assumptions. First, it assumes no memory in the system. Second, it assumes that radiation is caused by the state itself rather than the change of this state (once an actor is sick it has a chance to radiate). Capturing SIR/SIR models by implementing these two assumptions allow us to reduce equation 9 to:

$$p^{out}_{i,e,t} \sim Bern(p) \quad \text{in which} \quad p = s_{i,t} \times \alpha^*_{i,e,t} \quad \Longrightarrow \tag{12}$$

$$p^{out}_{i,e,t} = \begin{cases} \sim Bern(\alpha^*_{i,e,t}) & \text{if} \quad s_{i,t} = 1 \\ 0 & \text{if} \quad s_{i,t} = 0 \end{cases} \tag{13}$$

Combining this with the notion that in stochastic processes the incoming signals are of size 1 or 0 ($p^{out}_{i,e,t} \in \{1, 0\}$) and that the latter case is a trivial as there is nothing to propagate allows for rewriting transmission in these settings as:

$$p^{in}_{e,j,t} \sim Bern(\phi^*_{e,t}) \quad \text{if} \quad p^{out}_{i,e,t} = 1 \tag{14}$$

And using the same logic ($p^{in}_{e,j,t} \in \{1, 0\}$) the reception sub-process for the SIS/SIR models can be rewritten as:

$$\Delta s_{j,t} = \begin{cases} \sim Bern(1 - \prod (1 - \Psi^*_{j,t})) & \text{if} \quad s_{i,t} = 0 \\ 0 & \text{if} \quad s_{i,t} = 1 \end{cases} \tag{15}$$

Generalizability of the RTR-model

The previous paragraph shows that the RTR-model can be easily adjusted to mimic SIS/SIR like propagation models. However, as previously indicated, there is a variety of models for studying propagation: The SIS type of models (discussed above), bass [2] type of models, threshold type of models [3, 4] and cascade models [5]. Each of model types can be considered as special case of the binary RTR-model.

Bass-like RTR-model

Bass-like models [2] consider propagation to be a function of the previous adoption and a default adoption likelihood on a system level. It argues that a focal actors probability to adopt (read reception) is a function of the *proportion* of previous adopters. This can be interpreted as a special case of the reception process in which the reception of actors is a function of the signals coming from *all* actors in the network. In order to be able to receive signals from all actors this effectively assumes a fully connected network graph underlying the propagation process.
The Bass model does not differentiate between the three sub-processes and hence does not make specific claims about radiation or transmission. From the way the model is described it can be substantiated that this type of model assumes that the state of all actors in the system is known. Effectively, this implies that any actor in the adopted (infected) state radiates a signal by default and that this signal is transmitted by default as well. Consequently all other actors are 'informed' about this state. In terms of the three sub-processes these Bass-like models hence assume radiation to be a function of the state, and a 100% chance to radiate. It can hence be formalized in a similar form as the SIS model:

$$p^{out}_{i,e,t} \sim Bern(p) \quad \text{in which} \quad p = s_{i,t} \times \alpha^*_{i,t} \quad \Longrightarrow \tag{16}$$

$$p^{out}_{i,e,t} = \begin{cases} \sim Bern(\alpha^*_{i,e,t}) & \text{if} \quad s_{i,t} = 1 \\ 0 & \text{if} \quad s_{i,t} = 0 \end{cases} \tag{17}$$

In which it is assumed that $\alpha^*_{i,e,t} = 1$.

Similarly the transmission process is the result of the signals radiated and is assumed to always be successful, consequently any signal coming in will result in a signal going out. Transmission can hence be formulated the same way as in the SIS model:

$$p^{in}_{e,j,t} \sim Bern(\phi^*_{e,t}) \quad \text{if} \quad p^{out}_{i,e,t} = 1 \tag{18}$$

In which it is assumed that $\phi^*_{e,t} = 1$.

The reception sub process is only in part similar to the SIS scenario. The first part of the bass model considers imitation, the influence of previous adopters. It is a

function of the signals incoming, which given the completely connected graph underlying the propagation includes signals from all other actors in the system. This would imply a formulation can be used similar to the SIS model. However, as previously discussed bass-like models also capture innovation, which assumes there is also the default probability of adoption without these incoming signals.

There are two ways in which this secondary effect can be added. First, one could add a extra independent part to the reception function (this is what Bass did in his original model). This would however add an addition parameter to the model, but there is a more elegant solution. One could also introduce an additional control vertex in the graph. This vertex, like all other vertices, would be connected to all other vertices in the system. By making the reception likelihood for all edges coming from this control vertex independent of the other signals an additional 'random' adoption force can be introduced. The benefit of this approach is that the logic and formulations applied in the SIS setting apply to the Bass model as well, and hence reception in bass-like models can be formulated as:

$$\Delta s_{i,t} = \begin{cases} \sim Bern(1 - \prod 1 - \Psi^*_{i,t}) \quad \text{if} \quad s_{i,t} = 0 \\ 0 \quad \text{if} \quad s_{i,t} = 1 \end{cases} \tag{19}$$

By adjusting the $\eta_{i,e,t} \in \Psi^*_{i,t}$ for the edges coming from the control vertex the influence of the default adoption effect can be varied. One could even vary this across alters, hence such formulation directly increases the flexibility of the traditional bass-like models.

Threshold RTR-model of propagation

The threshold type of models (e.g. [4]) assume a somewhat different type of process dynamics. These models assume that the adoption occurs after a certain proportion of the connected alters have previously adopted. Literature on threshold models commonly limits itself to describing an adoption (read reception) process, implicitly assuming (similar to the previously introduced Bass models) that radiation and transmission will take place by default. Therefore the radiation and transmission functions can be assumed to be the same as in the previously considered Bass model (equation 16 and

18).

Unlike the previously discussed bass (and SIS) models threshold models are deterministic in nature. While this has no implication for the formulation of the radiation and transmission sub-processes (as they already are assumed to always occur) it does change the way the reception is formulated. This process should be deterministic rather than stochastic. Note that the general model of propagation was in fact deterministic, and hence the binary state version of this model (equation 8) describes such a process already. As the formulation of the general model already included the notion of limited susceptibility to signals and hence included a threshold the threshold model is already captured by the binary state version of the RTR-model. The only additional assumption being made is that the reception threshold of each actor (q_i) is a function of its number of neighbors ($E^{in}_{i,t}$)

Cascade RTR-model of propagation

The last general type of propagation model, the cascade models [5], is different from the previously discussed models. It can be considered as a special case of the stochastic binary RTR-model. In cascade models an external perturbation is considered which consequently can result in a cascading dynamics. Like in the binary RTR-model this perturbation changes the state of one (or more) vertices to 'active' (or infected) and consequently this change in activity will cascade to all of its alters (read radiate) with certain probability of success. The logic in this process is similar to the one described in the stochastic binary radiation process (equation 9). Key assumption in the cascading models is however that a vertex will only try to activate its alters once, effectively incorporating the constraint of no memory in the radiation process. Therefore the equation 9 can be simplified, and the radiation for a cascade model can be described as:

$$p^{out}_{i,e,t} \sim Bern(p) \quad \text{in which} \quad p = \Delta s_{i,t} \times \alpha^*_{i,e,t} \tag{20}$$

As the cascade models assume a setting without recovery ($\Delta s_{i,t} \in \{0, 1\}$) a constraint should be added to the equation, resulting in:

$$p^{out}_{i,e,t} = \begin{cases} p^{out}_{i,e,t} \sim Bern(\alpha^{*}_{i,e,t}) & \text{if} \quad \Delta s_{i,t} = 1 \\ 0 & \text{if} \quad \Delta s_{i,t} = 0 \end{cases} \tag{21}$$

The other sub-processes are not completely neglected in the cascade models. While there is no specific mention of transmission, it is assumed that each signal radiated will lead to a change in state of the alter. Consequently this type of model assumes that both transmission and reception will always occur. Transmission can therefore be formulated as:

$$p^{out}_{i,e,t} = p^{in}_{e,j,t} \quad \text{with} \quad e = (i,j) \tag{22}$$

While in cascades models the assumption is that radiation will result in propagation, neglecting the reception sub-process, these models do explicitly mention what happens when multiple changes in state would occur in a alters neighborhood (implicitly suggesting there is some reception sub-process). In such cases cascading models assume that each signal is processed sequentially (in a random order). As such it can be formalized as:

$$\Delta s_{j,t} = Bern(1 - \prod_{e \in E^{in}_{i},t} (1 - (Bern(\eta_{e,i,t} \times p^{in}_{e,i,t})))) \tag{23}$$

While the order of calculations is different compared to the RTR version —each incoming signal separately has a success chance, and then the product of success per signal is taken, versus, combining the signals (taking the product) and then consider the success chance of the combination— the fact that the general RTR-model assumes no distortion during aggregation (simply uses the product) means these functions coincide.

References

1. Kermack WO, McKendrick AG. Contributions to the mathematical theory of epidemics-I. Bulletin of mathematical biology. 1991;53(1-2):33–55.

2. Bass FM. A New Product Growth for Model Consumer Durables. Management Science. 1969;15(5):215–227. doi:10.1287/mnsc.15.5.215.

3. Rogers EM. Diffusion of Innovations, Fourth Edition. 4th ed. New York: Free Press; 1995.

4. Valente TW. Social network thresholds in the diffusion of innovations. Social Networks. 1996;18(1):69–89. doi:10.1016/0378-8733(95)00256-1.

5. Kempe D, Kleinberg J, Tardos v. Maximizing the Spread of Influence Through a Social Network. In: Proceedings of the Ninth ACM SIGKDD International Conference on Knowledge Discovery and Data Mining. KDD '03. New York, NY, USA: ACM; 2003. p. 137–146. Available from: http://doi.acm.org/10.1145/956750.956769.

6. Pastor-Satorras R, Vespignani A. Epidemic dynamics and endemic states in complex networks. Physical Review E. 2001;63(6):066117. doi:10.1103/PhysRevE.63.066117.

7. Pastor-Satorras R, Vespignani A. Epidemic Spreading in Scale-Free Networks. Physical Review Letters. 2001;86(14):3200–3203. doi:10.1103/PhysRevLett.86.3200.

8. Pastor-Satorras R, Castellano C, Van Mieghem P, Vespignani A. Epidemic processes in complex networks. Reviews of Modern Physics. 2015;87(3):925–979. doi:10.1103/RevModPhys.87.925.

9. Vermeer W. Propagation in Networks: The impact of information processing at the actor level on system-wide propagation dynamics; 2015. Available from: https://www.erim.eur.nl/doctoral-programme/phd-in-management/phd-projects/detail/678-competing-in-smart-business-networks-processes-of-value-creation-and-

10. Goldenberg J, Muller E. Using Complex Systems Analysis to Advance Marketing Theory Development: Modeling Heterogeneity Effects on New Product Growth through Stochastic Cellular Automata. Academy of Marketing Science Review; Vancouver. 2001;2001:1.
11. Kleinberg J. Cascading behavior in networks: Algorithmic and economic issues. In: Algorithmic game theory. Cambridge University Press; 2007. p. 613–632.
12. Centola D, Macy M. Complex Contagions and the Weakness of Long Ties. American Journal of Sociology. 2007;113(3):702–734. doi:10.1086/521848.
