# Supplementary material for: The Radiation-Transmission-Reception (RTR) model of propagation: Implications for the effectiveness of network interventions
Source: PLoS One. 2018 Dec 5;13(12):e0207865. doi: 10.1371/journal.pone.0207865 (PMC6281238; doi:10.1371/journal.pone.0207865)
Supplement: S4 File — (PDF) [file pone.0207865.s004.pdf]

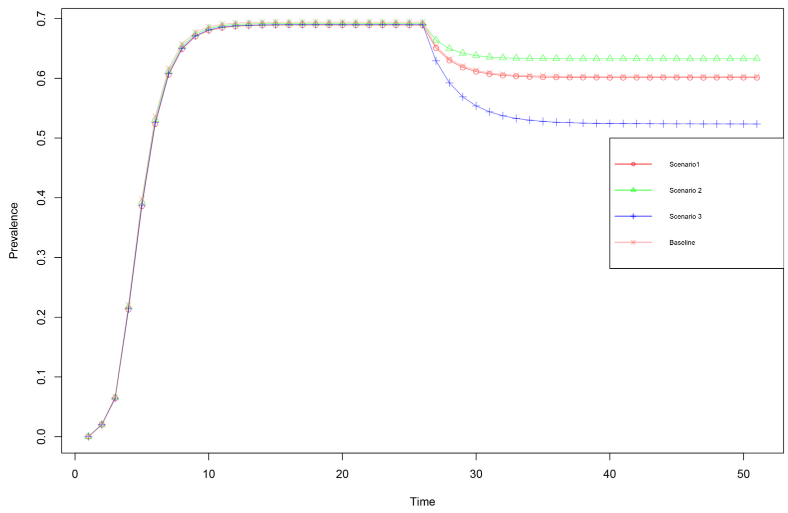

A. The mean prevalence over time for network 1 with a random seeding strategy is depicted for the four scenarios with the same overarching propagation likelihood ($\lambda = 0.192$). Scenario1, the red open circles, is a process with $\alpha = 0.4$, $\phi = 0.6$ and $\eta = 0.8$. Scenario2, the green triangles, a process with $\alpha = 0.6$, $\phi = 0.8$ and $\eta = 0.4$. Scenario3, blue pluses, a process with $\alpha = 0.8$, $\phi = 0.4$ and $\eta = 0.6$. Scenario 4, the pink crosses, a process with homogeneous sub-processes ($\alpha = \phi = \eta = 0.5769$). During the simulation at t = 25 an intervention (reduction of 0.2) in the Transmission is applied.

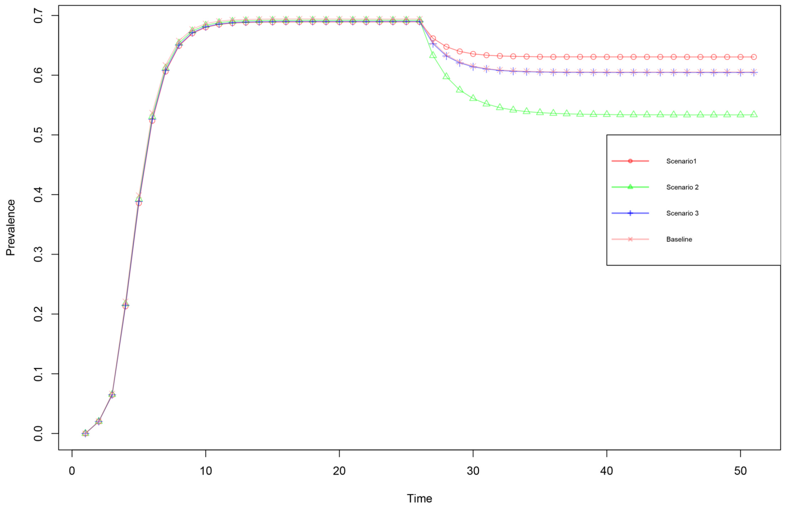

B. The mean prevalence over time for network 1 with a random seeding strategy is depicted for the four scenarios with the same overarching propagation likelihood ($\lambda = 0.192$). Scenario1, the red open circles, is a process with $\alpha = 0.4$, $\phi = 0.6$ and $\eta = 0.8$. Scenario2, the green triangles, a process with $\alpha = 0.6$, $\phi = 0.8$ and $\eta = 0.4$. Scenario3, blue pluses, a process with $\alpha = 0.8$, $\phi = 0.4$ and $\eta = 0.6$. Scenario 4, the pink crosses, a process with homogeneous sub-processes ($\alpha = \phi = \eta = 0.5769$). During the simulation at t = 25 an intervention (reduction of 0.2) in the Reception is applied.
