# Supplementary material for: The Radiation-Transmission-Reception (RTR) model of propagation: Implications for the effectiveness of network interventions
Source: PLoS One. 2018 Dec 5;13(12):e0207865. doi: 10.1371/journal.pone.0207865 (PMC6281238; doi:10.1371/journal.pone.0207865)
Supplement: S5 File — (PDF) [file pone.0207865.s005.pdf]

As previously indicated both the network structure and seed chosen can have strong effects on the propagation dynamics. As we aim to show the impact of the mechanism of propagation both of these effects have been controlled for during in the results presented in the main manuscript. However to make sure our results generalize this section will vary these dimensions, as discussed in our experimental design, and discuss our finding.

Network structures

In the main text the results of the synthetically generated scale-free network have been presented. However, as discussed in our experimental design, a similar set of experiments has also been conducted on two additional real world networks. The first, a Facebook Ego-network [1], which covers a giant component of friendship ties, based on the social circles on Facebook platform, with $n = 4.039$ and $e = 88.234$. The second, the Autonomous systems AS-733 network [2], which describes the communication among a set of autonomous systems, and is based on BGP (Border Gateway Protocol) logs, with 6.474 nodes and 12.572 edges. The results of these simulations are presented in Table A in which network 1 is the the original synthetically generated network, network 2 is the Facebook network, and network 3 is the AS-733 network. This table provides various insights into the impact of network structure. For example by considering the no-intervention scenarios one can observe that the level of prevalence at equilibrium varies across network structures, clearly supporting the role of network structure on propagation dynamics found in a wide range of previous studies. Similarly, in line with previous work in the propagation domain, by comparing any of the interventions across networks it becomes evident that the effectiveness of interventions (both relative and absolute) strongly varies and is conditional upon the network structure.

However, most importantly for the value of the RTR model: when the impact of the decomposition (the relative difference from the baseline scenario) is compared across networks, one finds that the effect of decomposing the process into the Radiation-Transmission-Reception sub-processes on network interventions is robust across networks. This indicates that regardless of the network structure, the decomposition of the mechanism plays a consistent role in determining intervention effectiveness.

A. This table provides an overview of all intervention effects, for each scenario and each network.

Scenario	Intervention	Unperturbed prevalence			Reduction in prevalence due to the intervention (the relative effect)			Difference in Intervention effect compared to the baseline scenario 4		
		Network 1	Network 2	Network 3	Network 1	Network 2	Network 3	Network 1	Network 2	Network 3
**1**	**None**	0.697	0.793	0.055	-	-	-	-	-	-
**2**	**None**	0.697	0.793	0.055	-	-	-	-	-	-
**3**	**None**	0.697	0.793	0.055	-	-	-	-	-	-
**4**	**None**	0.697	0.793	0.055	-	-	-	-	-	-
**1**	**Rad**	0.697	0.793	0.055	0.156 (22.4%)	0.039 (5.0%)	0.017 (30.5%)	176.5%	189.6%	165.0%
**2**	**Rad**	0.697	0.793	0.055	0.084 (12.0%)	0.020 (2.5%)	0.010 (17.6%)	94.6%	95.2%	94.9%
**3**	**Rad**	0.697	0.793	0.055	0.057 (8.2%)	0.013 (1.6%)	0.007 (12.2%)	64.6%	61.7%	66.7%
**4**	**Rad**	0.697	0.793	0.055	0.088 (12.7%)	0.021 (2.6%)	0.010 (18.5%)	100.0%	100.0%	100.0%
**1**	**Rec**	0.697	0.793	0.055	0.057 (8.1%)	0.013 (1.6%)	0.007 (12.2%)	64.1%	62.4%	66.2%
**2**	**Rec**	0.697	0.793	0.055	0.156 (22.4%)	0.039 (5.0%)	0.017 (30.5%)	175.9%	190.0%	165.2%
**3**	**Rec**	0.697	0.793	0.055	0.084 (12.0%)	0.019 (2.5%)	0.010 (17.6%)	94.6%	93.7%	95.1%
**4**	**Rec**	0.697	0.793	0.055	0.089 (12.7%)	0.021 (2.6%)	0.010 (18.5%)	100.0%	100.0%	100.0%
**1**	**Tra**	0.697	0.793	0.055	0.084 (12.0%)	0.020 (2.5%)	0.010 (17.6%)	94.5%	95.1%	95.1%
**2**	**Tra**	0.697	0.793	0.055	0.057 (8.2%)	0.013 (1.6%)	0.007 (12.2%)	64.3%	63.1%	66.4%
**3**	**Tra**	0.697	0.793	0.055	0.156 (22.4%)	0.039 (5.0%)	0.017 (30.5%)	176.1%	190.5%	165.7%
**4**	**Tra**	0.697	0.793	0.055	0.088 (12.7%)	0.021 (2.6%)	0.010 (18.5%)	100.0%	100.0%	100.0%

Seeding Strategy

It is known that the chosen seed can have a substantial impact on propagating dynamics of processes. To control for the potential impact of the chose seed two seeding strategies are explored. Random seeding has been used as the default strategy, in this strategy a single seed is randomly selected for each simulation. The second strategy explored is labeled the betweenness strategy, in this strategy seeding is 'optimized' by selecting the actor with the highest betweenness centrality as the seed in each simulation. It is often considered as one of the more basic and intuitive targeted seeding strategies. A third strategy called LeaderRank [3], in which the K-coreness of the network is leveraged, and the actor with the highest LeaderRank [3] is selected as the seed for each simulation, has also been explored. However, for our experimental setting, which uses one single seed to initialize the propagation process, it was found that the LeaderRank seeding strategy resulted in exactly the same optimal seed as betweenness. This is not to say that LeaderRank and betweennees will always results in the same seeding strategy, most likely the strategies will diverge once a seeding strategy based on multiple seeds is taken into account. Yet for our experimental setup both strategies where found them to give identical results. As most nuanced seeding strategies aim to at least partially leverage the betweenness, in cases with single seed such strategies are likely to similarly rank the highest betweenness node as the top influencer. Consequently, such strategies yield the same results as the betweenness strategy, and hence we restrict our comparison to two strategies, Fig. B presents this comparison. While it describes one specific scenario and intervention (Scenario 1 under a radiation intervention) the plot is indicative of the results overall (Table C), as the plots for the other sub-processes and interventions show an identical picture. These results indicate that the chosen seeding strategy impacts the rate at which propagation occurs only in the early stages, the dynamics equilibrium in prevalence remains unchanged. As in our experimental design interventions are implemented at a stage where the dynamic equilibrium has been reached, we find that the seeding strategy does not affect the intervention outcomes.

C. This table provides an overview of the differences between the seeding strategies for all scenarios and interventions in network 1.

Network	Scenario	Intervention	Seedtype	Unperturbed prevalence	Ending Prevalence	Intervention Effect
1	1	None	Random	0.697	0.697	0.000
1	1	None	Betweenness	0.697	0.697	0.000
1	2	None	Random	0.697	0.697	0.000
1	2	None	Betweenness	0.697	0.697	0.000
1	3	None	Random	0.697	0.697	0.000
1	3	None	Betweenness	0.697	0.697	0.000
1	4	None	Random	0.697	0.697	0.000
1	4	None	Betweenness	0.697	0.697	0.000
1	1	Rad	Random	0.697	0.541	0.156
1	1	Rad	Betweenness	0.697	0.541	0.156
1	2	Rad	Random	0.697	0.613	0.084
1	2	Rad	Betweenness	0.697	0.613	0.083
1	3	Rad	Random	0.697	0.640	0.057
1	3	Rad	Betweenness	0.697	0.640	0.057
1	4	Rad	Random	0.697	0.608	0.088
1	4	Rad	Betweenness	0.697	0.608	0.088
1	1	Rec	Random	0.697	0.640	0.057
1	1	Rec	Betweenness	0.697	0.640	0.057
1	2	Rec	Random	0.697	0.541	0.156
1	2	Rec	Betweenness	0.697	0.541	0.156
1	3	Rec	Random	0.697	0.613	0.084
1	3	Rec	Betweenness	0.697	0.613	0.084
1	4	Rec	Random	0.697	0.608	0.089
1	4	Rec	Betweenness	0.697	0.608	0.088
1	1	Tra	Random	0.697	0.613	0.084
1	1	Tra	Betweenness	0.697	0.613	0.084
1	2	Tra	Random	0.697	0.640	0.057
1	2	Tra	Betweenness	0.697	0.640	0.057
1	3	Tra	Random	0.697	0.541	0.156
1	3	Tra	Betweenness	0.697	0.541	0.156
1	4	Tra	Random	0.697	0.608	0.089
1	4	Tra	Betweenness	0.697	0.608	0.088

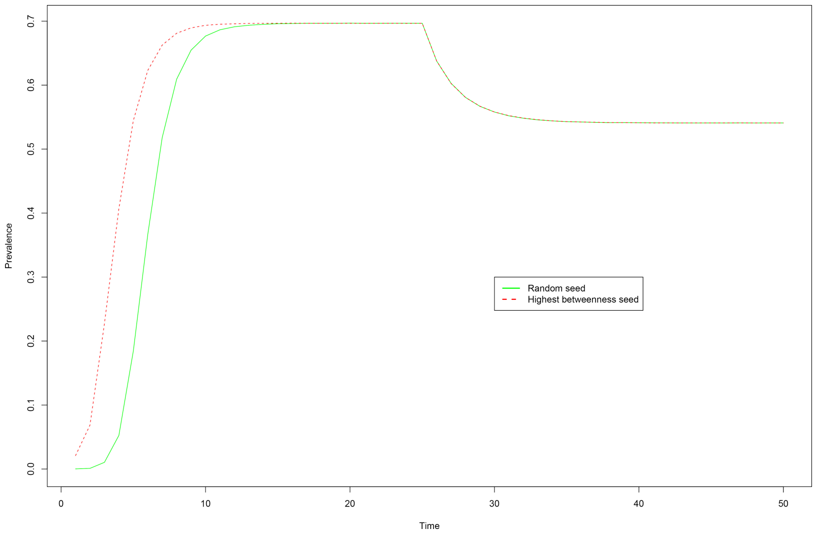

Fig B. This figure shows the mean prevalence (y-axis) in scenario 1 ($\alpha = 0.4$, $\phi = 0.6$ and $\eta = 0.8$) in network 1 with an intervention in Radiation under two seeding strategies. The green solid line shows the prevalence for the random seeding strategy, while the dashed red line describes the betweenness seeding strategy.

References

1. McAuley J, Leskovec J. Learning to Discover Social Circles in Ego Networks. In: Advances in Neural Information Processing Systems; 2012.

2. Leskovec J, Kleinberg J, Faloutsos C. Graphs over Time: Densification Laws, Shrinking Diameters and Possible Explanations. ACM SIGKDD International Conference on Knowledge Discovery and Data Mining (KDD); 2005.

3. Lü L, Zhang Y, Yeung C, Zhou T. Leaders in Social Networks, the Delicious Case. Plos ONE; 2011,6(6). doi10.1371/journal.pone.0021202.
