# Supplementary material for: The Radiation-Transmission-Reception (RTR) model of propagation: Implications for the effectiveness of network interventions
Source: PLoS One. 2018 Dec 5;13(12):e0207865. doi: 10.1371/journal.pone.0207865 (PMC6281238; doi:10.1371/journal.pone.0207865)
Supplement: S6 File — (PDF) [file pone.0207865.s006.pdf]

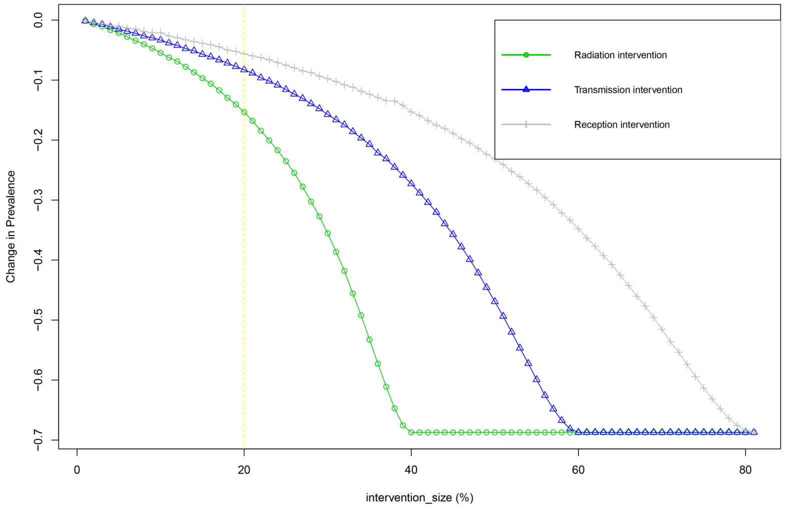

A. The mean reduction in prevalence (y-axis) in scenario 1 ($\alpha = 0.4$, $\phi = 0.6$ and $\eta = 0.8$) in network 1, with random seeding, for interventions of various size(x-axis) across the different target processes, green circles for interventions in Radiation (α), blue triangles for interventions in transmission (ϕ), and gray pluses for interventions in reception (η)

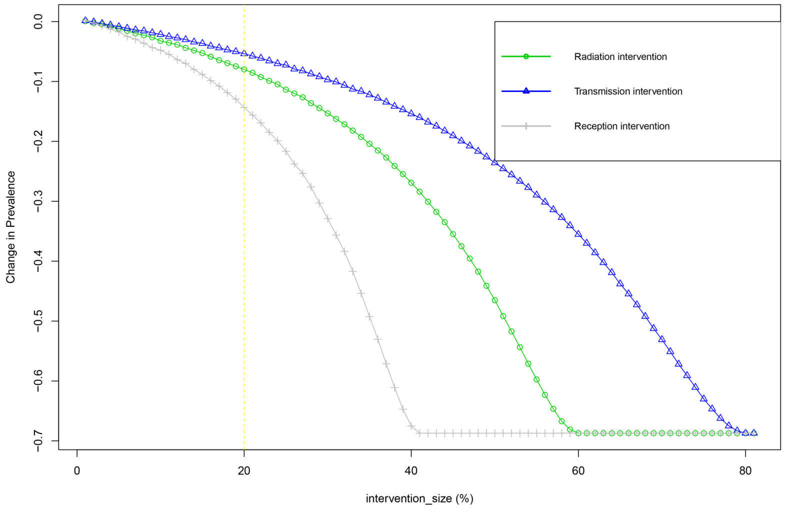

B. The mean reduction in prevalence (y-axis) in scenario 2 ($\alpha = 0.6$, $\phi = 0.8$ and $\eta = 0.4$) in network 1, with random seeding,for interventions of various size(x-axis) across the different target processes, green circles for interventions in Radiation (α), blue triangles for interventions in transmission (ϕ), and gray pluses for interventions in reception (η)
