# Supplementary material for: The Radiation-Transmission-Reception (RTR) model of propagation: Implications for the effectiveness of network interventions
Source: PLoS One. 2018 Dec 5;13(12):e0207865. doi: 10.1371/journal.pone.0207865 (PMC6281238; doi:10.1371/journal.pone.0207865)
Supplement: S7 File — (ZIP) [file pone.0207865.s007.zip › Output-plots/Net2Int4Seed2.pdf]

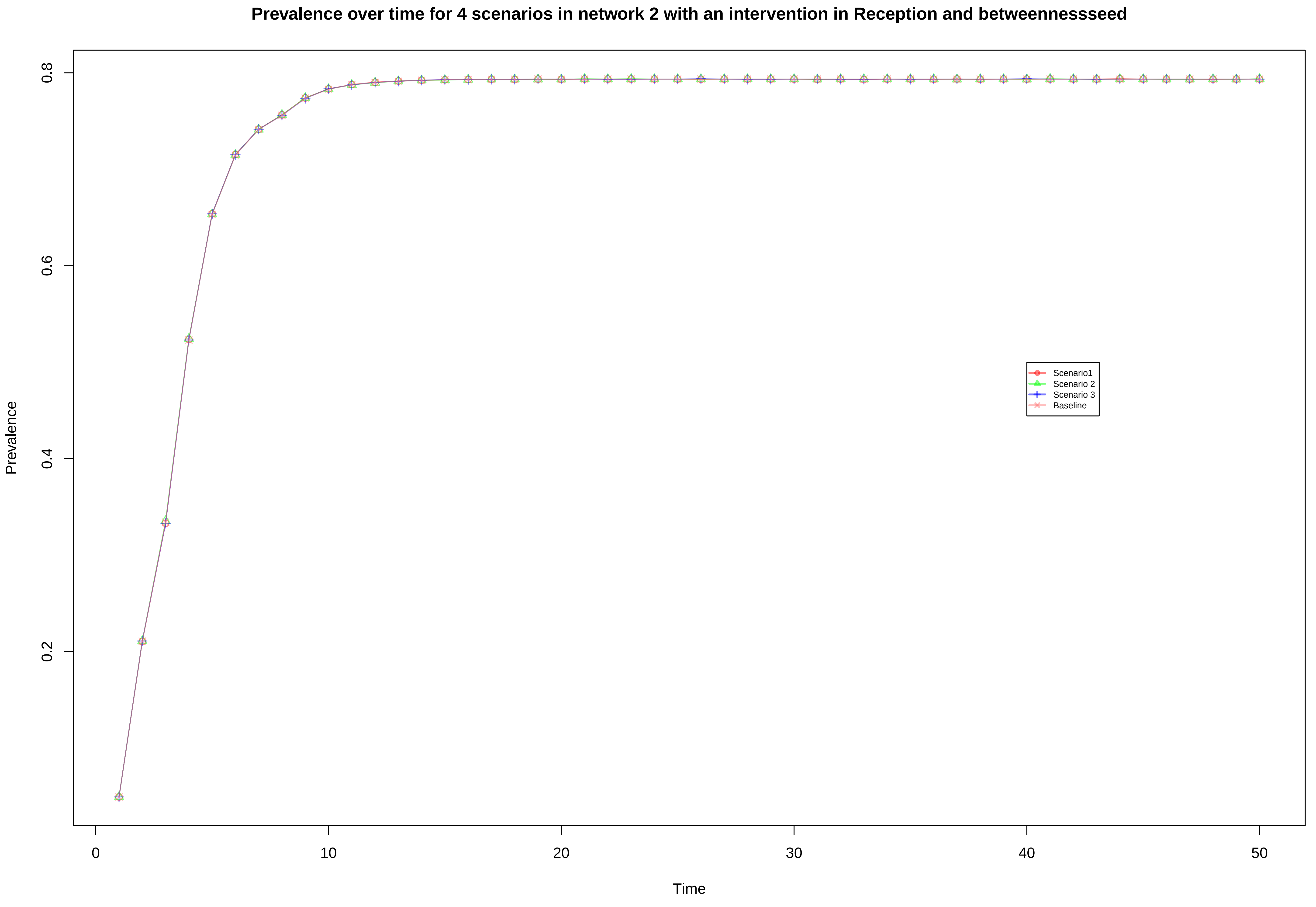
Prevalence over time for 4 scenarios in network 2 with an intervention in Reception and betweennessseed
Prevalence
0.2
0.4
0.6
0.8
Time
0
10
20
30
40
50
Scenario1
Scenario 2
Scenario 3
Baseline
