# Supplementary figures and images for: The Radiation-Transmission-Reception (RTR) model of propagation: Implications for the effectiveness of network interventions
Source: PLoS One. 2018 Dec 5;13(12):e0207865. doi: 10.1371/journal.pone.0207865 (PMC6281238; doi:10.1371/journal.pone.0207865)

Prevalence over time for 4 scenarios in network 1 Randomly seeded without intervention

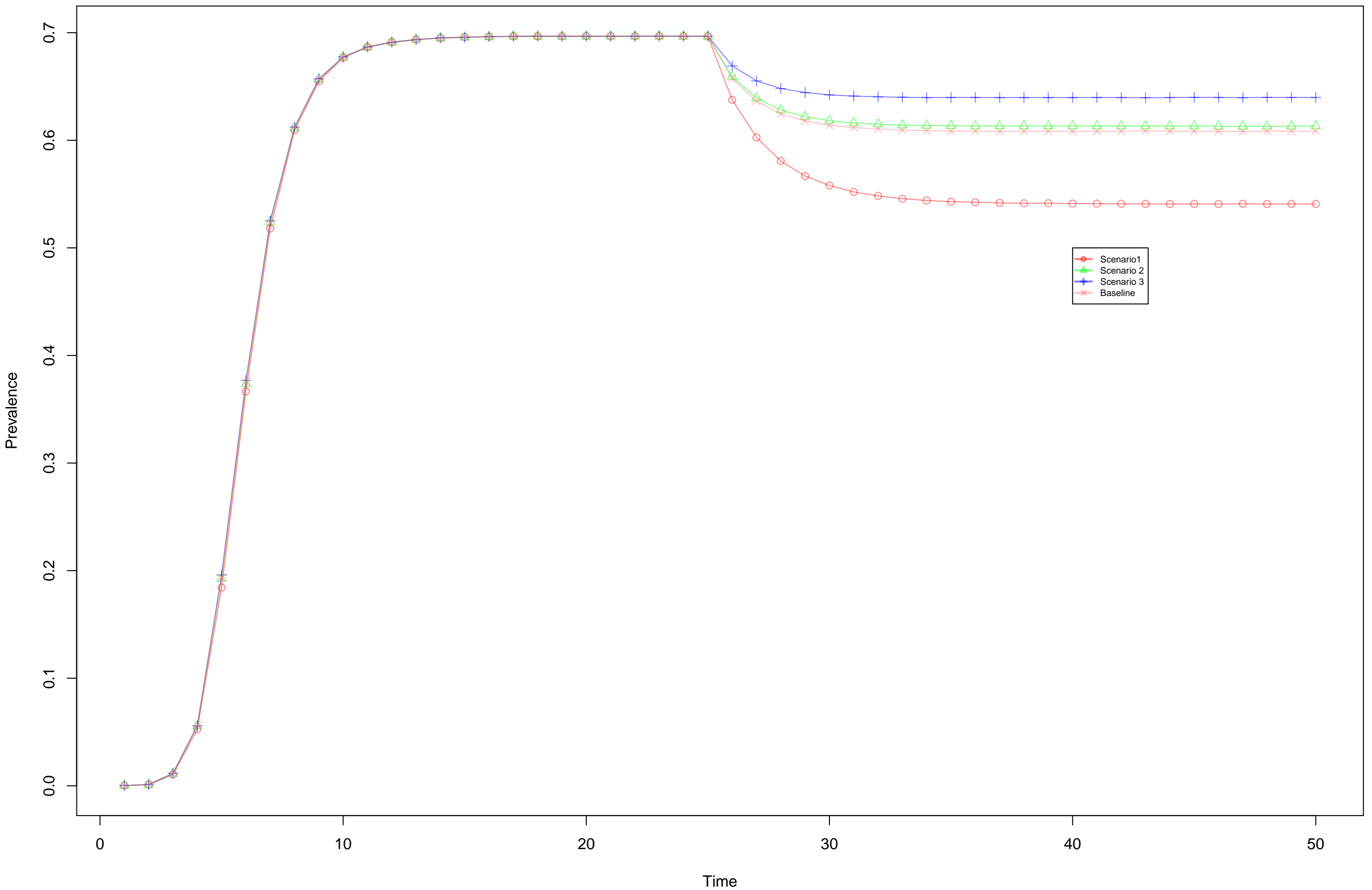

Supplement: S7 File — (ZIP) [file pone.0207865.s007.zip › Output-plots/Net1Int1Seed1.pdf]

Prevalence over time for 4 scenarios in network 1 Randomly seeded without intervention

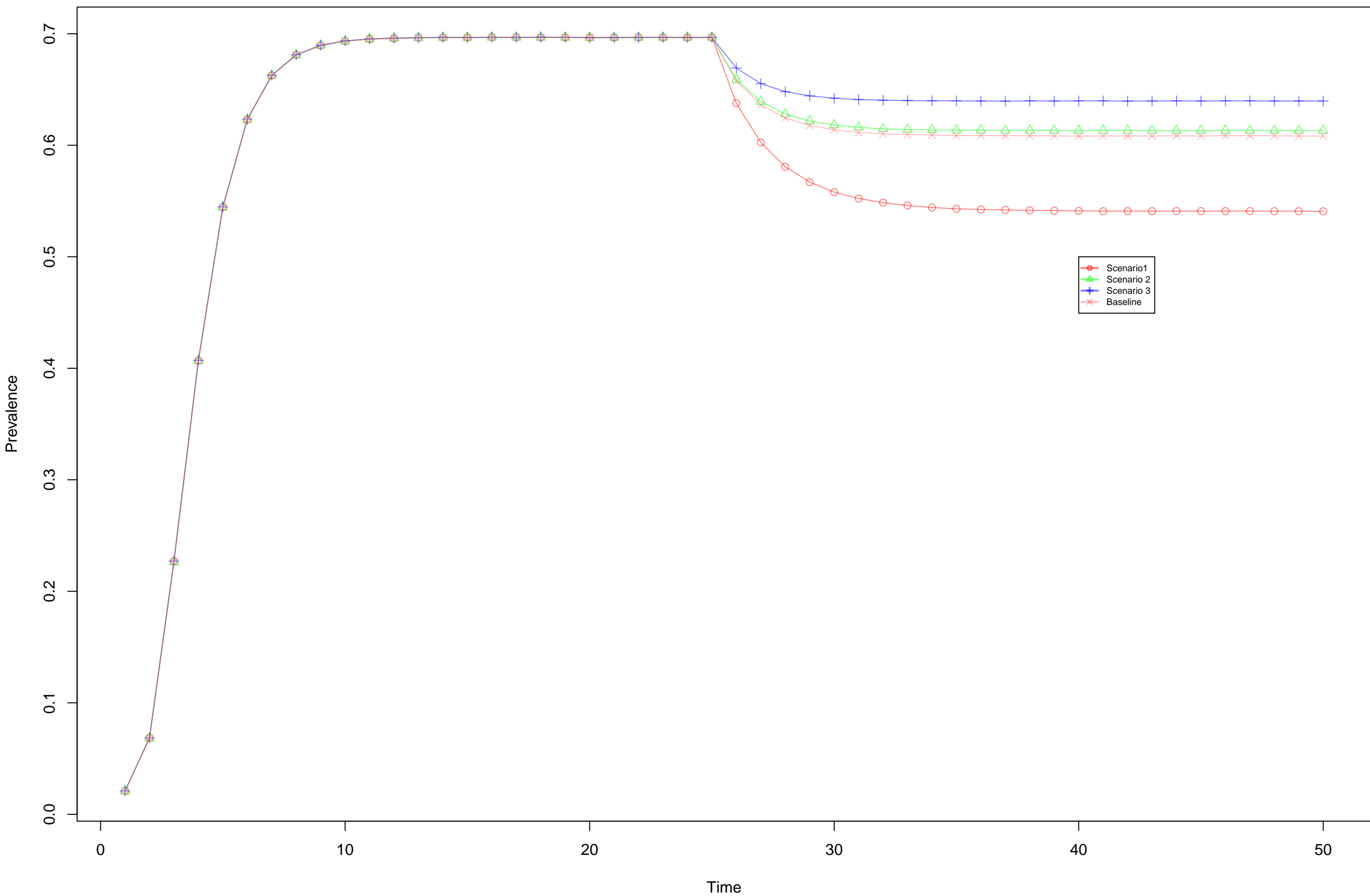

Supplement: S7 File — (ZIP) [file pone.0207865.s007.zip › Output-plots/Net1Int1Seed2.pdf]

Prevalence over time for 4 scenarios in network 2 Randomly seeded without intervention

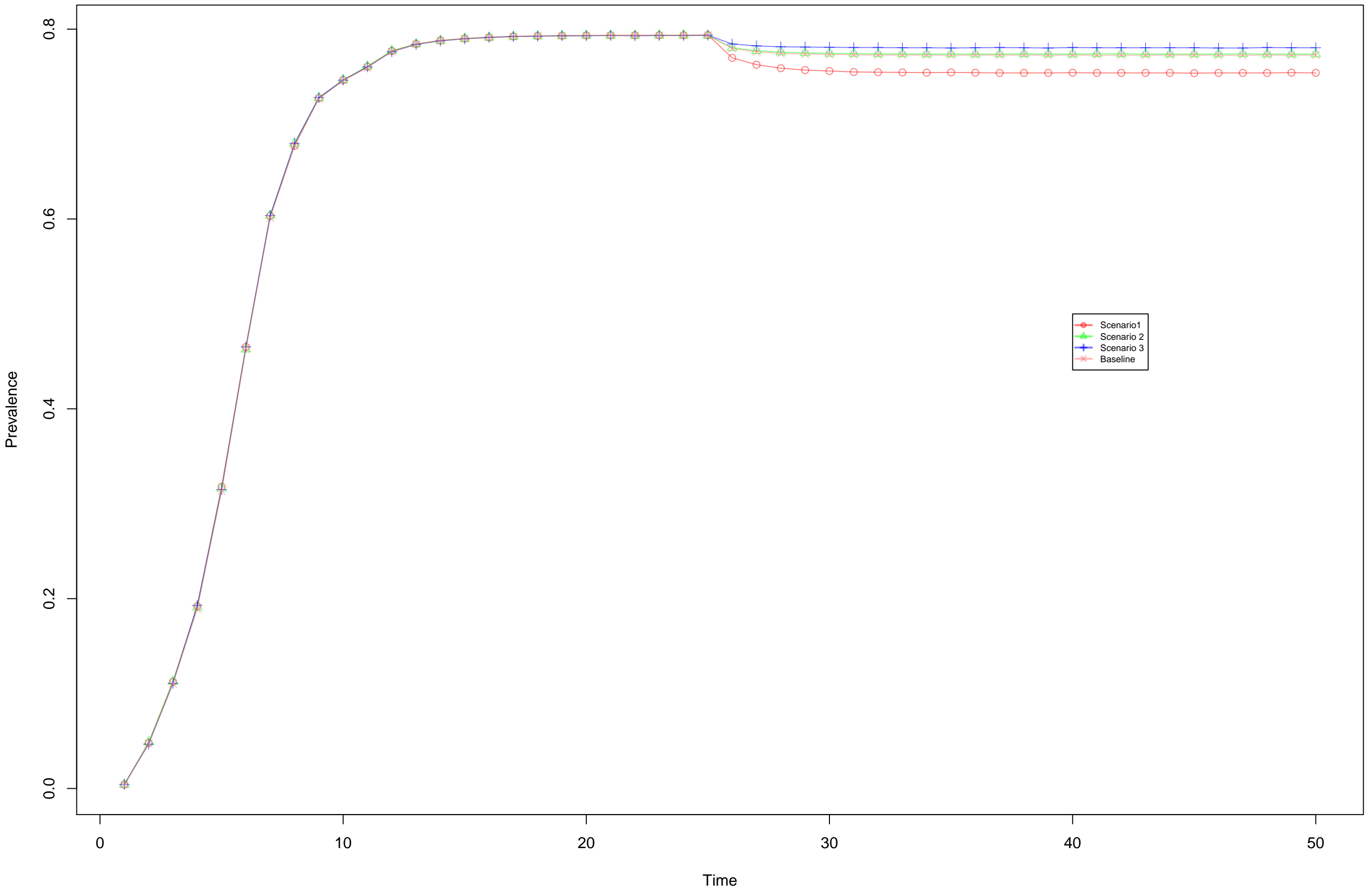

Supplement: S7 File — (ZIP) [file pone.0207865.s007.zip › Output-plots/Net2Int1Seed1.pdf]

Prevalence over time for 4 scenarios in network 2 Randomly seeded without intervention

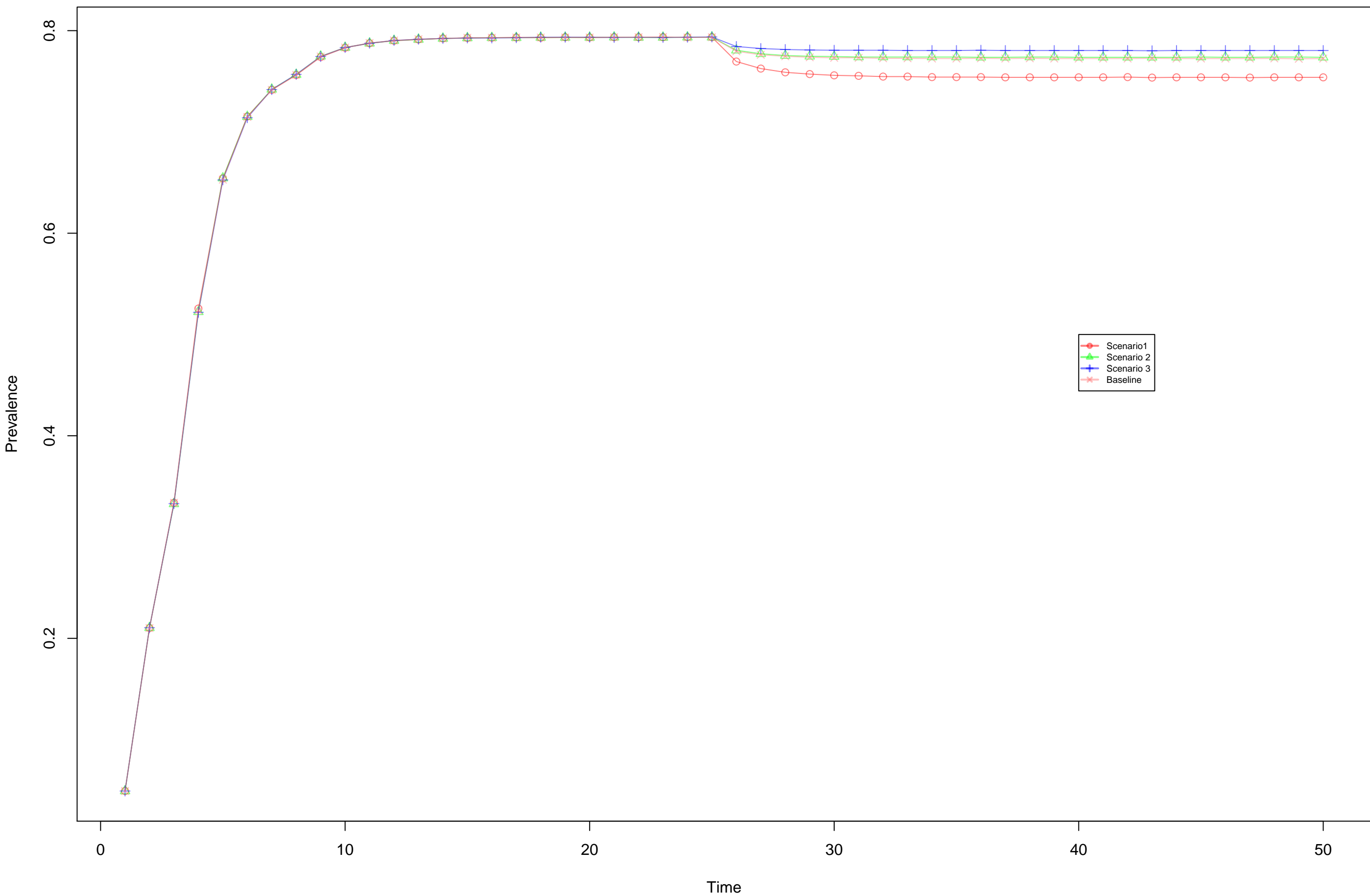

Supplement: S7 File — (ZIP) [file pone.0207865.s007.zip › Output-plots/Net2Int1Seed2.pdf]

Prevalence over time for 4 scenarios in network 3 Randomly seeded without intervention

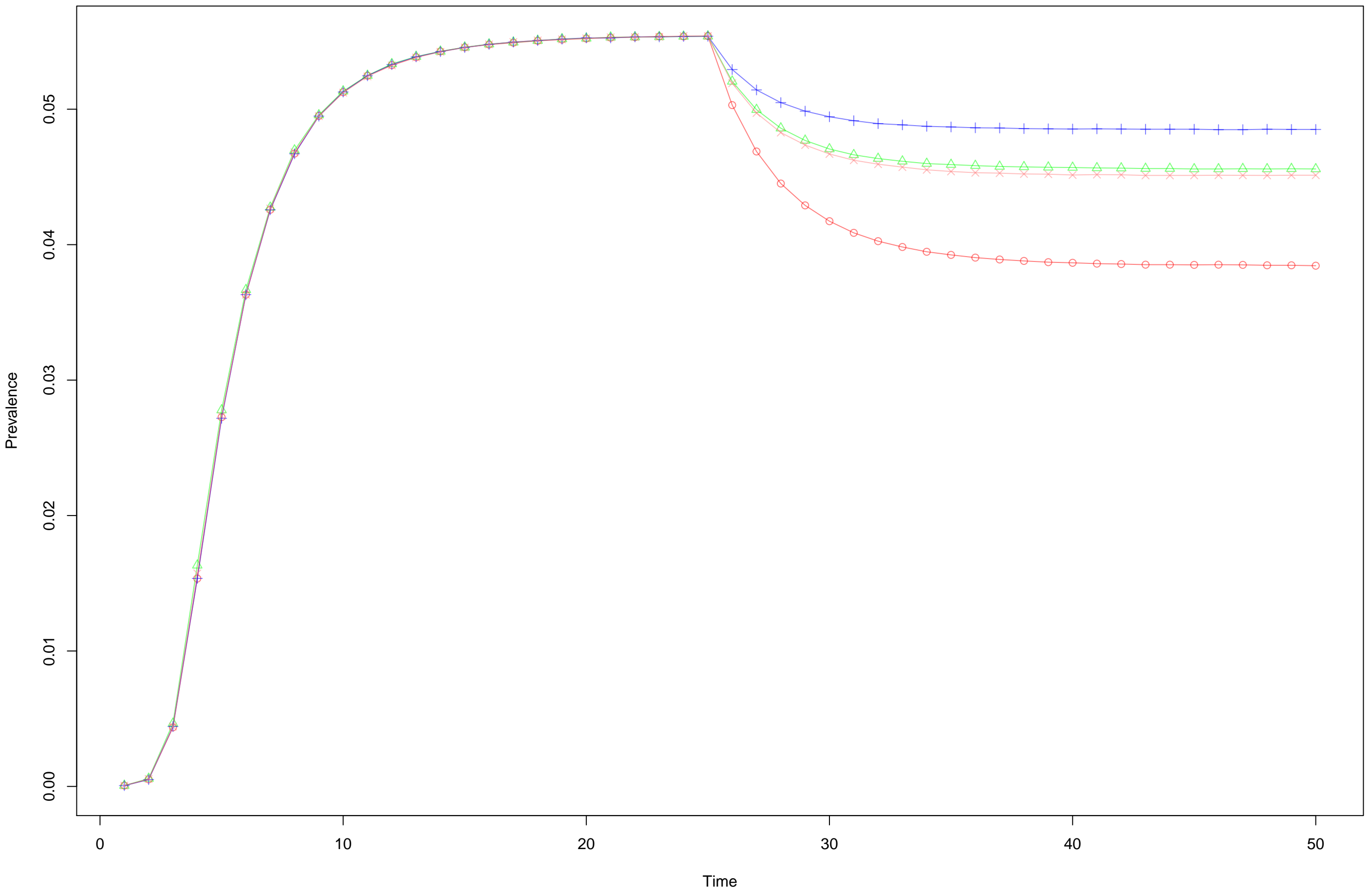

Supplement: S7 File — (ZIP) [file pone.0207865.s007.zip › Output-plots/Net3Int1Seed1.pdf]

Prevalence over time for 4 scenarios in network 3 Randomly seeded without intervention

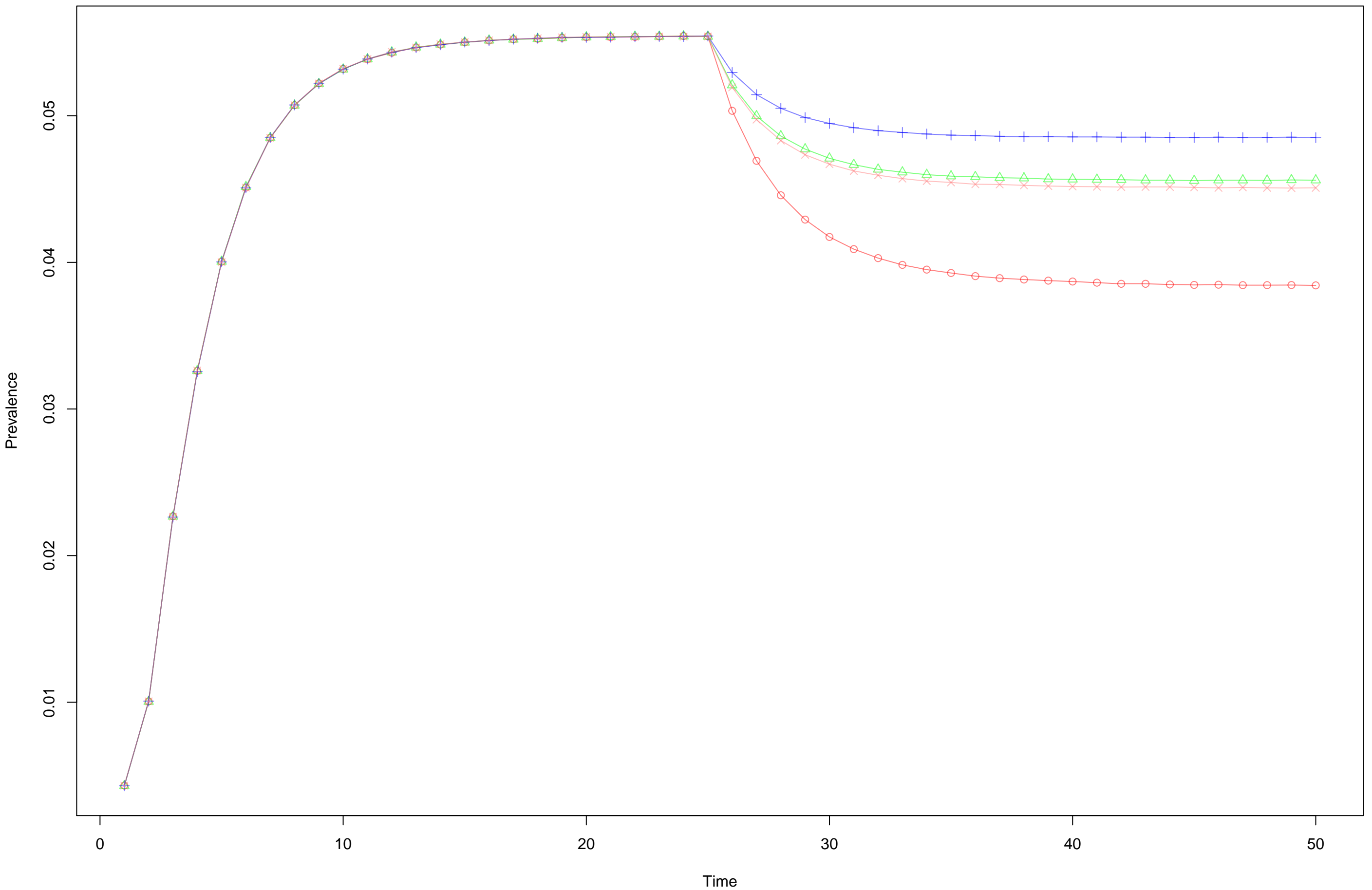

Supplement: S7 File — (ZIP) [file pone.0207865.s007.zip › Output-plots/Net3Int1Seed2.pdf]
